# Kidins220 deficiency causes ventriculomegaly via SNX27-retromer-dependent AQP4 degradation

**DOI:** 10.1038/s41380-021-01127-9

**Published:** 2021-05-17

**Authors:** Ana del Puerto, Julia Pose-Utrilla, Ana Simón-García, Celia López-Menéndez, Antonio J. Jiménez, Eva Porlan, Luis S. M. Pajuelo, Guillermo Cano-García, Beatriz Martí-Prado, Álvaro Sebastián-Serrano, Marina P. Sánchez-Carralero, Fabrizia Cesca, Giampietro Schiavo, Isidro Ferrer, Isabel Fariñas, Miguel R. Campanero, Teresa Iglesias

**Affiliations:** 1 Instituto de Investigaciones Biomédicas “Alberto Sols”, Consejo Superior de Investigaciones Científicas-Universidad Autónoma de Madrid (CSIC-UAM), Madrid, Spain; 2 Centro de Investigación Biomédica en Red de Enfermedades Neurodegenerativas (CIBERNED), Instituto de Salud Carlos III, Madrid, Spain; 3 Departamento de Biología Celular, Genética y Fisiología, Facultad de Ciencias, Universidad de Málaga, Bulevar Louis Pasteur, Málaga, Spain; 4 Instituto de Investigación Biomédica de Málaga (IBIMA), Instituto de Salud Carlos III, Málaga, Spain; 5 Centro de Biología Molecular “Severo Ochoa” (CSIC-UAM), Madrid, Spain; 6 Departamento de Biología Molecular, Facultad de Ciencias, UAM, Madrid, Spain; 7 Instituto de Investigación Hospital Universitario La Paz (IdiPAZ), Instituto de Salud Carlos III, Madrid, Spain; 8 Departmento de Biología Celular, Biología Funcional y Antropología Física, Universidad de Valencia, Burjassot, Spain; 9 Department of Life Sciences, University of Trieste, Trieste, Italy; 10 Department of Neuromuscular Disorders, UCL Institute of Neurology, University College London, London, UK; 11 UK Dementia Research Institute, University College London, London, UK; 12 Departamento de Patología y Terapéutica Experimental, Universidad de Barcelona, Barcelona, Spain; 13 Instituto de Neurociencias, Universidad de Barcelona, Hospitalet de Llobregat, Barcelona, Spain; 14 Servicio de Anatomía Patológica, Hospital Universitario de Bellvitge, Carrer de la Feixa Llarga, Barcelona, Spain; 15 Centro de Investigación Biomédica en Red en Enfermedades Cardiovasculares (CIBERCV), Instituto de Salud Carlos III, Madrid, Spain; 16 Present Address: Departmento de Biotecnología, Instituto Nacional de Investigación y Tecnología Agraria y Alimentaria (INIA), Madrid, Spain; 17 Present Address: Departmento de Bioquímica y Biología Molecular, Facultad de Veterinaria, Universidad Complutense de Madrid, Madrid, Spain

**Keywords:** Schizophrenia, Neuroscience, Cell biology

## Abstract

Several psychiatric, neurologic and neurodegenerative disorders present increased brain ventricles volume, being hydrocephalus the disease with the major manifestation of ventriculomegaly caused by the accumulation of high amounts of cerebrospinal fluid (CSF). The molecules and pathomechanisms underlying cerebral ventricular enlargement are widely unknown. *Kinase D interacting substrate of 220* *kDa* (*KIDINS220)* gene has been recently associated with schizophrenia and with a novel syndrome characterized by spastic paraplegia, intellectual disability, nystagmus and obesity (SINO syndrome), diseases frequently occurring with ventriculomegaly. Here we show that Kidins220, a transmembrane protein effector of various key neuronal signalling pathways, is a critical regulator of CSF homeostasis. We observe that both KIDINS220 and the water channel aquaporin-4 (AQP4) are markedly downregulated at the ventricular ependymal lining of idiopathic normal pressure hydrocephalus (iNPH) patients. We also find that Kidins220 deficient mice develop ventriculomegaly accompanied by water dyshomeostasis and loss of AQP4 in the brain ventricular ependymal layer and astrocytes. Kidins220 is a known cargo of the SNX27-retromer, a complex that redirects endocytosed plasma membrane proteins (cargos) back to the cell surface, thus avoiding their targeting to lysosomes for degradation. Mechanistically, we show that AQP4 is a novel cargo of the SNX27-retromer and that Kidins220 deficiency promotes a striking and unexpected downregulation of the SNX27-retromer that results in AQP4 lysosomal degradation. Accordingly, SNX27 silencing decreases AQP4 levels in wild-type astrocytes whereas SNX27 overexpression restores AQP4 content in Kidins220 deficient astrocytes. Together our data suggest that the KIDINS220-SNX27-retromer-AQP4 pathway is involved in human ventriculomegaly and open novel therapeutic perspectives.

## Introduction

Hydrocephalus is a disease associated with cognitive impairment where cerebrospinal fluid (CSF) accumulates provoking brain ventricular enlargement or ventriculomegaly. There are multiple hydrocephalus forms, including foetal, neonatal, paediatric and adult onset presentation, and diverse aetiologies such as genetic and developmental factors, viral infections, co-morbidities and aging [1–4]. Although hydrocephalus is the pathology that reaches maximal brain ventricles enlargement, ventriculomegaly is also a co-morbidity factor in several psychiatric and neurodegenerative disorders, including schizophrenia [5, 6], Parkinson´s disease [7] and Alzheimer´s disease (AD) [8]. Dementia is one of the characteristic symptoms of chronic hydrocephalus in adults, where the major form is idiopathic normal pressure hydrocephalus (iNPH), a disease recently proposed to be neurodegenerative with dysfunctions shared with AD [9–11].

There is an urgent need to identify and characterize the pathogenic mechanisms governing CSF dynamics and underlying brain ventricular enlargement in different neuropathologies to uncover novel therapeutic strategies. Very little is known about the molecules involved in the cellular processes that contribute to ventriculomegaly, although the water selective channel aquaporin-4 (AQP4) appears as a critical player. AQP4 expression in the brain is prominent in the basolateral membranes of ependymocytes lining cerebral ventricles and in the end-feet of astrocytes contacting brain blood vessels and the pia membrane [12, 13]. *Aqp4*^*−/−*^ mice show sporadic brain ventricular enlargement, accelerated progression of induced hydrocephalus, and increased basal brain water accumulation [14–18], suggesting that AQP4 is critical for controlling whole-brain water homeostasis (reviewed in [11, 19–21]). While studies in iNPH brain revealed loss of astrocytic perivascular AQP4 [22, 23], the underlying mechanisms of AQP4 downregulation remain unknown. It is also unknown whether AQP4 content at the ependymal ventricular lining is altered in iNPH.

The recycling of endocytosed transmembrane proteins (cargos) from early endosomes back to the plasma membrane might be involved in ventriculomegaly development, as suggested by the presence of severe postnatal hydrocephalus in mice lacking sorting nexin 27 (*Snx27*) [24]. SNX27 associated with vacuolar protein sorting 35 (VPS35), VPS29 and VPS26 forms the so-called SNX27-retromer, a complex specifically involved in the endosomal-to-plasma membrane recycling of selected cargos, that are degraded at lysosomes upon SNX27 loss [25, 26]. Importantly, retromer impairment also contributes to aberrant endocytic trafficking in various neurodegenerative diseases [27].

Kinase D interacting substrate of 220 kDa (Kidins220 [28]), also known as ankyrin-repeat rich membrane spanning (ARMS [29]), is a SNX27-retromer cargo targeted to lysosomal degradation in the absence of this PDZ protein [25]. Kidins220 is an effector of several signalling pathways, including those downstream neurotrophin and glutamate N-methyl-D-aspartate (NMDA) receptors, and modulates neuronal differentiation and synaptic activity [30, 31]. Neuronal survival depends on Kidins220 and the expression of this molecule is altered in neurological and neurodegenerative disorders, including cerebral ischemia, AD and Huntington´s disease [32–36]. In addition, rare novel missense variants in *KIDINS220* gene have been associated with schizophrenia [37–39], a psychiatric disorder with a strong linkage to ventriculomegaly [5, 6]. More recently, *KIDINS220* nonsense and loss-of-function variants have been associated with SINO syndrome, a novel rare autosomal disease characterized by spastic paraplegia, intellectual disability, nystagmus and obesity, signs that concur with different degrees of ventriculomegaly [40, 41]. Here we identify Kidins220 as a key determinant in the control of brain water homeostasis, ventricular enlargement and hydrocephalus pathology by molecular mechanisms that involve the unexpected expression regulation of SNX27-retromer components, which in turn controls AQP4 turnover.

## Materials and methods

### Human brain samples

Brain samples from iNPH patients (*n* = 6) and non-normotensive hydrocephalus patients (*n* = 3), and control subjects (*n* = 8) (for data see Supplementary Table 1) were obtained from the Institute of Neuropathology Brain Bank (IDIBELL, Hospitalet de Llobregat, Spain), tissue Biobank from Hospital Clinic de Barcelona and Institute d´Investigacions Biomediques August Pi I Sunyer (HCB-IDIBAPS Biobank), and the Biobank Banco de Tejidos CIEN (PT17/0015/0014), integrated into the Spanish National Biobanks Network following Spanish legislation and local Ethics Committee guidelines and processed following standard operating procedures as described [42]. Briefly, one brain hemisphere was fixed by immersion in 4% buffered formalin for 3 weeks. Samples of selected regions of the brain were embedded in paraffin and 15-μm sections were stained for neuropathological studies using immunohistochemistry methods (see ‘Immunohistochemistry of mouse and human brain samples’ section and details for antibodies and dilutions used in Supplementary Table 2). All participants gave their written consent, and the study was approved by the local Ethics and Scientific Committees following the ethical standards recommended by the Helsinki Declaration.

### Experimental animals

*Kidins220*^*f/f*^ mice and *Kidins220*^*−/−*^ mice in C57BL/6 J background had been previously generated in G. Schiavo laboratory [43]. Male and female 2-month-old *Kidins220*^*+/f*^, *Kidins220*^*f/f*^ or WT littermates were employed in independent experiments for comparison purposes. Genotyping was performed by PCR using specific pairs of primers [43]. All animals were produced and housed at the animal care facility at Instituto de Investigaciones Biomédicas ‘Alberto Sols’ (IIBM, CSIC-UAM, Madrid, Spain) and maintained under 12/12 h light-dark cycle and with access to food and water *ad libitum* in a temperature-controlled environment. Overall mouse health was assessed by daily inspection for signs of discomfort, weight loss or changes in behaviour, mobility and feeding or drinking habits. Procedures involving animals had been approved by Institutional (IIBM and CSIC) and local Ethical Committees, and were conformed to the appropriate national legislations (RD 53/2013) and the guidelines of the European Commission for the accommodation and care of laboratory animals (revised in Appendix A of the Council of Europe Convention ETS123).

Magnetic resonance imaging; Scanning electron microscopy; RNA isolation, reverse transcription and quantitative real-time PCR; Plasmids; Cell culture, treatment and transfection of primary cortical astrocytes and HEK293T cells; Lentiviral production and transduction of astrocytic cultures; Water permeability and cell-volume measurements in cultured astrocytes; Commercial antibodies; Preparation of protein extracts, immunoprecipitation and immunoblot analysis; Immunofluorescence of cultured astrocytes; Immunohistochemistry of mouse and human brain samples; Image acquisition and Quantitative and statistical analysis were performed as described in the Extended Data Supplementary Material and Methods section. Pairs of primers for quantitative real-time PCR and sh-oligonucleotides cloned in lentiviral vectors are detailed in Supplementary Table 3.

## Results

### *Kidins220* deficient mice present ventriculomegaly

**Fig. 1:**
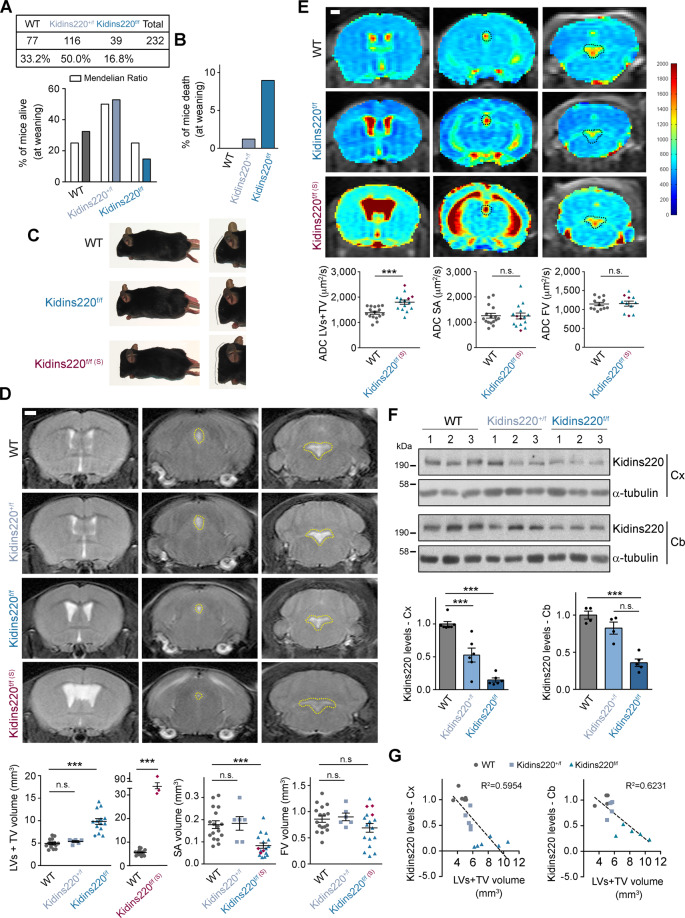
Ventriculomegaly in *Kidins220* hypomorphic mice. **A** Total number (*n* = 232) and percentage of WT, *Kidins220*^*f/+*^ and *Kidins220*^*f/f*^ mice alive at weaning *vs* their expected Mendelian ratio. **B** Percentage of WT, *Kidins220*^*f/+*^ and *Kidins220*^*f/f*^ mice death at weaning showing severe hydrocephalic phenotype (*n* = 232). **C** Representative images of 2-month-old WT, *Kidins220*^*f/f*^, and severely hydrocephalic *Kidins220*^*f/f*^ (Kidins220^f/f(S)^) mice. Right: Zoom detail of the head. **D** Representative in vivo T2-weighted (T2-W) MRI coronal images showing lateral and third ventricles (LVs + TV; left panels), aqueduct of Sylvius (SA; central panels—dotted line) and fourth ventricle (FV; right panels—dotted line) of 2-month-old WT, *Kidins220*^*f/+*^, *Kidins220*^*f/f*^ and *Kidins220*^*f/f(S)*^ male mice. Scale bar, 1 mm. Quantification of ventricular and aqueduct volume (mm^3^) in 17 WT (circles), 6 *Kidins220*^*f/+*^(squares) and 14 *Kidins220*^*f/f*^ (triangles) mice. LVs and TV values from the same group of 17 WT (circles) animals have been represented in a separate graph with those of three severe *Kidins220*^*f/f(S)*^ (diamonds) mice. **E** Apparent Diffusion Coefficient (ADC) representative coronal images showing LVs and TV (left panels), SA (central panels—dotted line) and FV (right panels—dotted line) of 2-month-old WT, *Kidins220*^*f/f*^ and *Kidins220*^*f/f(S)*^ male animals. The pseudo-colour scale indicates maximum and minimum ADC values in μm^2^/s. Scale bar, 1 mm. Quantification of ADC values (μm^2^/s) at the ventricles and aqueduct in WT (circles; LVs, TV and SA, *n* = 16; FV, *n* = 12), *Kidins220*^*f/f*^ (triangles; LVs, TV and SA, *n* = 12; FV, *n* = 8) and *Kidins220*^*f/f(S)*^ (*n* = 3, diamonds) mice. **F** Representative Kidins220 and α-tubulin (loading control) immunoblots of cortex (Cx) and cerebellum (Cb) lysates from WT, *Kidins220*^*f/+*^ and *Kidins220*^*f/f*^ mice; each lane represents one animal. Kidins220 levels are represented in arbitrary units after normalization with α-tubulin in *Kidins220*^*f/+*^ (Cx, *n* = 6; Cb, *n* = 4) and *Kidins220*^*f/f*^ (Cx, *n* = 6; Cb, *n* = 5) relative to WT (Cx, *n* = 7, Cb, *n* = 4) mice. **G** Correlation between Kidins220 relative levels in the cortex (Cx, left panel) and cerebellum (Cb, right panel) represented in (**F**) and LVs and TV volumes quantified in T2-W MRI images depicted in (**D**), established by linear regression analysis (Cx, R^2^: 0.5954; Cb, R^2^: 0.6231). **D**, **E**, **F** Data are mean ± s.e.m.; each data point denotes an individual mouse; *NS* not significant; ***P < 0.001, by one-way ANOVA (**D**, **F**) and two-tailed unpaired Student’s *t* test (**E**).

Because *Kidins220* complete ablation produces perinatal lethality, we created *Kidins220-floxed* mice to generate mouse models with a conditional deletion in different tissues and cells to study this molecule function in adulthood [43, 44]. We noticed that homozygous *Kidins220-floxed* mice (*Kidins220*^*f/f*^) were not present in the offspring of heterozygous (*Kidins220*^*+/f*^) crosses at Mendelian ratio, with a greater lethality at weaning (Fig. 1A, B). Two-month-old *Kidins220*^*f/f*^ animals did not show differences in body weight but presented an increment in brain weight compared to their littermates (Supplementary Fig. S1a). It was also visually apparent that a low percentage of *Kidins220*^*f/f*^ mice presented encephalomegaly (17.4%), referred to as ‘severe’ (S) (*Kidins220*^*f/f(S)*^), suggesting possible brain ventricular enlargement and hydrocephalus (Fig. 1C). MRI analysis confirmed the development of ventriculomegaly to various extents, from mild to severe, both in male and female *Kidins220*^*f/f*^ mice (Fig. 1D and Supplementary Fig. S1b). Volumetric quantification from T2-weighted (T2-W) images rendered increments in the volume of lateral ventricles (LVs) and third ventricle (TV) of male and female *Kidins220*^*f/f*^ animals compared to wild type (WT) or *Kidins220*^*+/f*^ littermates (Fig. 1D and Supplementary Fig. S1b). While no substantial changes were detected in the fourth ventricle (FV), the aqueduct of Sylvius (SA) volume was decreased in *Kidins220*^*f/f*^ males and females (Fig. 1D and Supplementary Fig. S1b). Using diffusion-weighted imaging MRI, we also calculated apparent diffusion coefficient (ADC) values as a measurement of the magnitude of diffusion of water molecules in the ventricles and SA of male mice. ADC values were elevated in LVs and TV from *Kidins220*^*f/f*^ mice relative to WT animals suggesting an imbalance in the circulation of CSF (Fig. 1E).

The generation of *Kidins220*^*f/f*^ animals involved genetic manipulations that integrated *Kidins220* cDNA within exon 16 of the mouse gene (see details in reference [43] and scheme in Supplementary Fig. S1c). We hypothesized that this strategy might potentially cause a deficiency in *Kidins220* expression. To test this hypothesis, we analysed Kidins220 levels in various brain regions. Peroxidase-based immunostaining of brain slices detected lower Kidins220 signal intensity in *Kidins220*^*f/f*^ compared to WT mice (Supplementary Fig. S1d). Immunoblot analysis showed that the cortex and cerebellum of *Kidins220*^*f/f*^ mice exhibited decreased amounts of Kidins220 compared to WT animals (Fig. 1F). The cortex of *Kidins220*^*+/f*^, but not the cerebellum, also presented substantially reduced levels of this protein, indicating that some brain regions are potentially more susceptible to the interference induced by the transgenic modification. Importantly, we found an inverse correlation between Kidins220 levels and LVs and TV ventricular volume in WT, *Kidins220*^*+/f*^, and *Kidins220*^*f/f*^ mice (Fig. 1G), suggesting that Kidins220 deficiency and brain ventricle enlargement are strongly linked. In addition to brain, extracts obtained from several tissues showed decreased levels of Kidins220 to various degrees between WT, *Kidins220*^*+/f*^, and *Kidins220*^*f/f*^ mice (Supplementary Fig. S1e). *Kidins220*^*f/f*^(s) mice were sacrificed immediately after MRI, and their brain tissue was not further analysed due to the high degree of damage (see MRI of a highly severe phenotype in Supplementary Fig. 2). Together, these data indicate that *Kidins220*^*f/f*^ mice constitute a hypomorphic model with marked deficiency of Kidins220 in several tissues, supporting the notion that the decrease of Kidins220 brain levels is linked to ventriculomegaly and hydrocephalus development.

### Loss of Kidins220 and AQP4 at the ventricular ependyma of *Kidins220* hydrocephalus mice and human iNPH patients

**Fig. 2:**
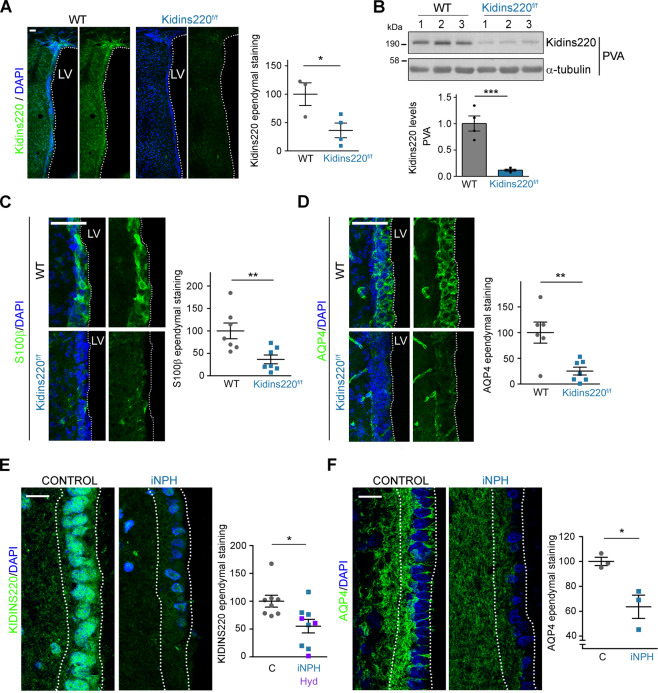
Decreased AQP4 and Kidins220 levels at the ependymal barrier of Kidins220 hypomorphic mice and human iNPH. **A** Representative confocal microscopy images of Kidins220 (green) and DAPI (blue) staining at the ependymal barrier of brain lateral ventricles (LV) of 2-month-old WT and *Kidins220*^*f/f*^ mice, and (right panel) quantification of Kidins220 immunostaining at the ependymal barrier of 4 *Kidins220*^*f/f*^ mice relative to 3 WT mice. Scale bar, 50 μm. **B** Kidins220 and α-tubulin (loading control) immunoblot analysis of the periventricular area (PVA) of WT and *Kidins220*^*f/f*^ mice (*n* = 4 per genotype); each lane represents one mouse. Kidins220 levels, after normalization with those for α-tubulin, were represented relative to WT mice (bottom panel). **C**, **D** Representative confocal microscopy images showing S100β (**C**), AQP4 (**D**) immunostaining (green) and DAPI nuclear labelling (blue) of lateral ventricles (LV) ependymal barrier from 2-month-old WT and *Kidins220*^*f/f*^ mice. Scale bar, 50 μm. Quantification of S100β (**C**; *n* = 7 animals per genotype) and AQP4 (**D**; *n* = 6 WT and *n* = 7 *Kidins220*^*f/f*^ mice) staining at the ependymal barrier of *Kidins220*^*f/f*^ mice relative to WT mice. **E**, **F** Representative confocal microscopy images of KIDINS220 (**E**) and AQP4 (**F**) immunostaining (green) and DAPI nuclear labelling (blue) at the ependymal barrier (dotted lines) of necropsies from iNPH patients and control subjects. Scale bars, 30 μm. Quantification of ependymal barrier immunostaining in human postmortem samples from hydrocephalic patients relative to control individuals: KIDINS220 (**E**) [iNPH *n* = 6; Hyd *n* = 3; control *n* = 8] and AQP4 (**F**) [iNPH *n* = 3; control *n* = 3]. For quantifications, three sections per animal and genotype and 3 ROIs per section (**A**, **C**, **D**), and 9 randomly selected ROIs per human section (**E**, **F**) were used; data are mean ± s.e.m.; each data point denotes an individual mouse/subject. (**A–F**) *0.01 < *P* < 0.05, **0.001 < *P* < 0.01, ***P < 0.001; by two-tailed unpaired Student’s *t* test.

Due to the importance of the ventricular ependyma in brain water homeostasis and congenital hydrocephalus [3], we examined the presence of Kidins220 in this specialised tissue by using a recently generated antibody [45]. Its specificity in immunofluorescence and immunoblot analyses was further validated by antibody neutralization with the immunizing peptide and by using brain tissue from *Kidins220*^*−/−*^ mice [43, 44] (Supplementary Fig. S3a–c). Confocal microscopy analysis showed higher ependymal Kidins220 expression in the lateral ventricles of WT mice compared to *Kidins220*^*f/f*^ mice (Fig. 2A). Accordingly, Kidins220 levels were significantly lowered in extracts prepared from the lateral periventricular area (PVA) of *Kidins220*^*f/f*^ animals (Fig. 2B).

The ependymal barrier lines cerebral ventricles and is formed by multiciliated ependymocytes that beat ventricular CSF synchronously to drive its unidirectional flow across ventricles [46, 47]. Different types of congenital hydrocephalus present developmental defects in ependymocytes differentiation, ciliogenesis and maturation, or denudation of the ependymal barrier [3, 48]. We, therefore, examined the morphological features of the ependymal cells in LVs of 2-month-old *Kidins220*^*f/f*^ mice by scanning electron microscopy, finding no apparent macroscopic changes in their cilia compared to WT animals (Supplementary Fig. S4a). In addition, organization and integrity of the ependymal barrier in *Kidins220* deficient mice was largely unaltered, as assessed by whole-mount preparation immunostaining and localization of β-catenin at the intercellular contacts and γ-tubulin at the base of the cilia (Supplementary Fig. S4b).

Although the ultrastructural appearance and planar polarity of the ependymal barrier in *Kidins220*^*f/f*^ animals were normal, ependymal cells lacked expression of S100 calcium-binding protein β (S100β), a protein expressed in ependymocytes and astrocytes [49] (Fig. 2C). We also examined levels of AQP4 in this area as this channel is also expressed in ependymal cells and plays a critical role in brain water homeostasis and ependyma function [19, 20]. *Kidins220*^*f/f*^ mice exhibited a striking downregulation of AQP4 in ependymal cells of LVs compared to WT animals, in immunofluorescence analysis of both brain sections and whole-mount preparations (Fig. 2D and Supplementary Fig. S4c). In contrast, no substantial changes in GFAP, a marker for neural stem cells and astrocytes, or FoxJ1 that marks mature ciliated ependymal cells [50, 51] were observed (Supplementary Fig. S4d, e).

Next, we examined potential decreases of KIDINS220 and AQP4 at the ventricular ependyma in iNPH patients compared to control donors (Supplementary Table 1). We found that ependymal cells were positive for KIDINS220 signal in brain necropsies from control individuals and that this staining diminished significantly in samples from equivalent brain regions from iNPH patients (Fig. 2E and Supplementary Fig. S5a–S6). Samples from adult non-normotensive hydrocephalus (Hyd) patients similarly presented a fainter KIDINS220 ependymal barrier signal than aged-matched controls (Fig. 2E and Supplementary Fig. S5a–S6). By contrast, S100β ependymal zone immunolabelling was unaltered in hydrocephalic versus control human samples (Supplementary Fig. S5b). Notably, AQP4 immunofluorescence analysis also showed a sharp signal drop in samples from iNPH patients (Fig. 2F and Supplementary Fig. S5c). This is the first evidence of an ependymal deficit of AQP4 and KIDINS220 in iNPH patients, supporting the involvement of the ependymal barrier and these two molecules in the development of adult chronic hydrocephalus.

### Downregulation of AQP4 in astrocytes from *Kidins220* hypomorphic mice

**Fig. 3:**
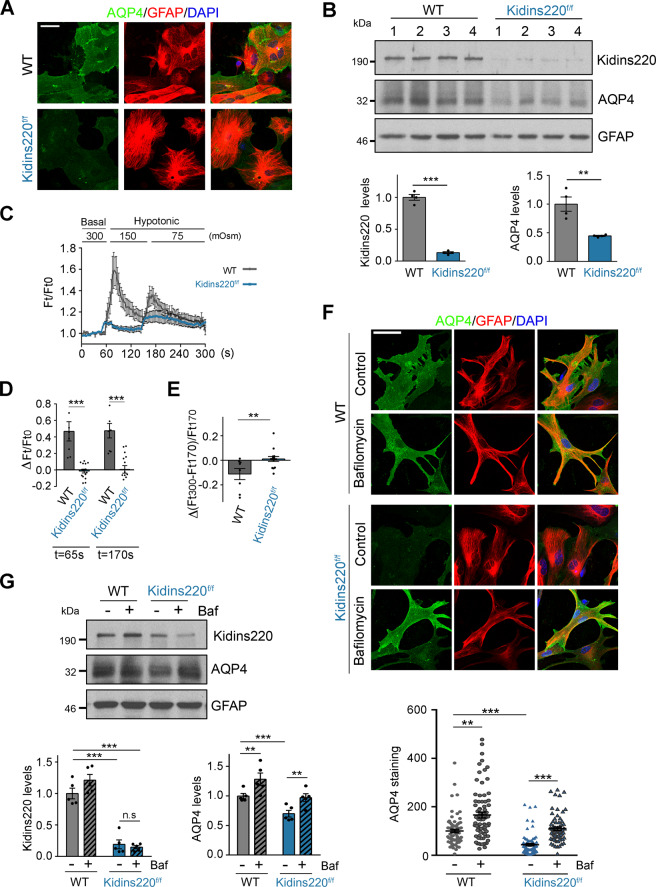
AQP4 downregulation in Kidins220-deficient astrocytes is rescued by inhibition of lysosomal degradation. **A** Representative confocal microscopy images of AQP4 (green), GFAP (red) and DAPI (blue) staining of primary cortical astrocytes dissected from WT (*n* = 3) and *Kidins220*^*f/f*^ (*n* = 3) mice. Scale bar, 50 μm. **B** (top panels) Kidins220, AQP4 and GFAP (loading control) immunoblot analyses of WT and *Kidins220*^*f/f*^ cortical astrocytes; each lane represents a culture obtained from one animal. (bottom panels) Kidins220 and AQP4 levels in *Kidins220*^*f/f*^ astrocytes expressed in arbitrary units after normalization with GFAP and relative to WT astrocytes (*n* = 4 of each genotype). **C**–**E** Primary astrocytes from WT (*n* = 3) and *Kidins220*^*f/f*^ (*n* = 3) mice were exposed to a hypotonic medium (ΔOsm = 150 and 75 mOsm) (*n* = 3 independent experiments); **C** Calcein-quenching measurement of osmotically induced volume changes in these cells (*n* = 6 of each genotype) during 300 s after a basal recording of 60 s. **D** Quantitative analysis of the initial volume increase osmotically induced in 7 WT and 14 *Kidins220*^*f/f*^ astrocytes from calcein-quenching measurement data; ordinate values represent mean volume changes at 65 s and 170 s (Ft) of hypotonic stress relative to WT baseline levels (Ft0). **E** Representation of the final volume osmotically induced changes in 8 WT and 12 *Kidins220*^*f/f*^ astrocytes from calcein-quenching measurement data; ordinate values represent mean volume changes incurred between the maximum volume at 170 s (Ft170) and the one registered at 300 s, at the end of the recording period (Ft300) to examine recovery capacity. See ‘Materials and Methods’ for quantification details. Regulatory volume decreases (RVD) indicative of volume recovery are represented by negative values. **F** Representative confocal microscopy images of AQP4 (green), GFAP (red) and DAPI (blue) staining of cultured astrocytes from WT and *Kidins220*^*f/f*^ mice incubated with vehicle (Control) or bafilomycin A1 for 16 h. Scale bar; 50 μm. Quantification of AQP4 immunostaining in GFAP-positive astrocytes under the indicated conditions; each data point represents a single astrocyte (*n* = 60–80 astrocytes per condition; *n* = 3 independent experiments). **G** Representative Kidins220, AQP4 and GFAP (loading control) immunoblot analysis of cultured cortical astrocytes incubated with vehicle (−) or bafilomycin A1 (Baf, +) obtained from WT and *Kidins220*^*f/f*^ mice. Quantification of Kidins220 and AQP4 levels after normalization with GFAP signal (*n* = 5 independent experiments) and relative to WT cells is shown (bottom panels). Quantification data are shown as mean ± s.e.m.; *NS* not significant; **0.001 < *P* < 0.01, ***P < 0.001; by two-tailed unpaired Student’s *t* test in (**B**, **D**, **E**) and two-way ANOVA in (**F**, **G**).

To gain mechanistic insight into the regulation of S100β and AQP4 expression by Kidins220, we analysed primary cultures of astrocytes because these cells contain high levels of these two molecules and, in contrast to ependymocytes, can be readily cultured, maintained and transfected/transduced in vitro. RTq-PCR showed no significant decreases of *Aqp4* or *S100β* mRNA in cultured astrocytes or in tissue from the PVA obtained from *Kidins220*^*f/f*^ animals (Supplementary Fig. S7). However, we observed a substantial decrease in Kidins220 and AQP4 in cultured astrocytes obtained from *Kidins220*^*f/f*^ mice (Fig. 3A, B), suggesting that post-transcriptional mechanisms underlie AQP4 downregulation. Of note, we could not detect S100β protein.

Astrocytes transiently increase their volume upon hypotonic stress and this process is regulated by AQP4 [52]. To determine whether AQP4 function is impaired in *Kidins220*^*f/f*^ astrocytes, we treated astrocytes with a hypotonic stimulus and measured their volume by using the calcein-quenching assay [52]. We found that *Kidins220*^*f/f*^ astrocytes did not increase their volume as much as WT cells upon hypotonic stress, and showed a significant delay in bringing their volume back to baseline (Fig. 3C–E). These results strongly suggest that AQP4 function is greatly hampered in Kidins220 deficient astrocytes.

We then investigated the potential post-transcriptional mechanisms involved in AQP4 downregulation. As this water channel can be degraded by the lysosome [53], we examined the effect of the vATPase inhibitor bafilomycin A1 on AQP4 levels. We found that bafilomycin A1 induced a significant increase in AQP4 levels both in WT and *Kidins220*^*f/f*^ astrocytes, as determined by immunofluorescence analysis and immunoblot quantification (Fig. 3F, G). Together, our data strongly suggest that Kidins220 deficiency increases AQP4 lysosomal degradation.

### Joint regulation of the SNX27-retromer and its novel cargo AQP4 by Kidins220

**Fig. 4:**
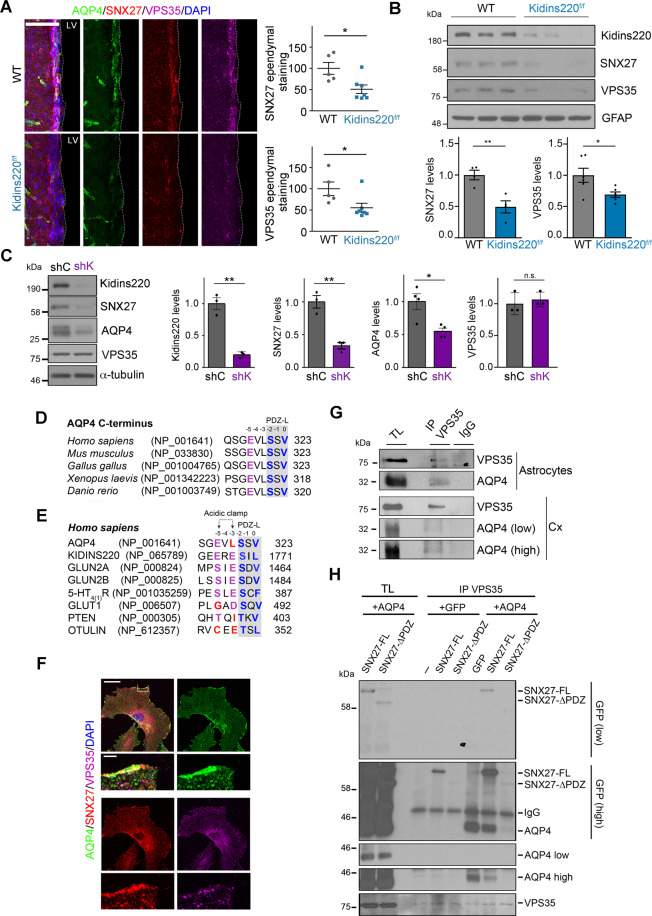
Joint regulation of the SNX27-retromer and its novel cargo AQP4 by Kidins220. **A** Representative confocal microscopy images of AQP4 (green), SNX27 (red), VPS35 (magenta) and DAPI (blue) staining of the ependymal barrier from lateral ventricles (LV) of WT (*n* = 5) and *Kidins220*^*f/f*^ (*n* = 7) mice; and (right panel) quantification of SNX27 and VPS35 staining of these samples (three sections per animal). Scale bar, 50 μm. **B** Kidins220, SNX27, VPS35 and GFAP (loading control) immunoblot analyses of WT and *Kidins220*^*f/f*^ cortical astrocytes; each lane represents a culture obtained from one animal. Graphs represent the quantification of SNX27 and VPS35 after normalization with GFAP. **C** Kidins220, SNX27, AQP4, VPS35 and α-tubulin (loading control) immunoblot analyses of cultured cortical astrocytes obtained from WT mice transduced with lentivirus encoding shControl (shC) or shKidins220 (shK). Quantification of the indicated protein levels after normalization with α-tubulin: Kidins220 (*n* = 3), SNX27 (*n* = 3), AQP4 (*n* = 4) and VPS35 (*n* = 3) independent experiments. **D** Sequence alignment of the last 9 amino acids within AQP4 C-terminal end of different organisms and their access number. **E** Sequence alignment of the last 8 amino acids of human AQP4 and the indicated known cargos of SNX27. PDZ-binding motif triplet residues (PDZ-L) are shown in grey boxes and critical amino acids at 0 and −2 position are labelled in blue. Negatively charged amino acids (aspartic and glutamic acid or phosphorylated serine, threonine residues) at −3 and −5 position enhancing SNX27 binding by forming an acidic clamp are shown in magenta. Note that in −3 position there is a leucine within AQP4 sequence or isoleucine within PTEN, and in the −5 position there is a glycine within GLUT1 and a cysteine within OCLUDIN (labelled in red). **F** Representative confocal microscopy images showing AQP4 (green), SNX27 (red), VPS35 (magenta) and DAPI (blue) staining in cultured WT astrocytes (*n* = 3 independent experiments). Scale bar, 50 μm. Zoom images from boxed regions are also shown. Scale bar, 5 μm. **G** Representative immunoprecipitation (IP) analysis of VPS35 in lysates from cortical cultured astrocytes or cortex (Cx) from WT mice and immunoblotting with VPS35 or AQP4 antibodies. Immunoprecipitation with a non-related immunoglobulin (IgG) was used as control. Total lysates (TL) were run in parallel (*n* = 3 independent experiments). Low and high exposure images of AQP4 in cortical lysates are included. **H** VSP35 was immunoprecipitated in lysates from HEK293T cells transfected with GFP or GFP-AQP4 and GFP-SNX27 full-length (SNX27-FL) or GFP SNX27 lacking the PDZ domain (SNX27-ΔPDZ). Representative immunoblot analysis of total lysates (TL) or VPS35-immunoprecipitates (IP VPS35) using GFP, AQP4 or VPS35 antibodies. Low and high exposures of GFP and AQP4 immunoblots are shown in order to analyse properly bands signal (*n* = 3 independent experiments). Quantification data are shown as mean ± s.e.m.; *NS* not significant, *0.01 < *P* < 0.05, **0.001 < *P* < 0.01; by two-tailed unpaired Student’s *t* test (**A**, **B**, **C**).

Given that Kidins220 endosomal recycling to the cell surface is controlled by the SNX27-retromer complex [25], and that *Snx27* genetic inactivation causes severe postnatal hydrocephalus in mice [24], we investigated whether hydrocephalus development in *Kidins220* deficient animals could be associated with SNX27-retromer dysfunction. Staining of VPS35, the key SNX27-retromer component [26], and SNX27 at the ependyma of LVs revealed a prominent decrease of their levels in *Kidins220*^*f/f*^ (Fig. 4A). Accordingly, astrocytes from *Kidins220*^*f/f*^ mice also showed a severe downregulation of SNX27 and VPS35 (Fig. 4B). We then checked whether Kidins220 could be directly involved in the control of SNX27 and VPS35 by transducing cultured astrocytes from WT mice with lentiviral particles encoding Kidins220 shRNA (shKidins220, referred as shK) or a control shRNA (shC). Strikingly, Kidins220 knockdown strongly reduced SNX27 protein levels without altering those of VPS35 (Fig. 4C). Consistent with results obtained in *Kidins220*^*f/f*^ astrocytes, Kidins220 silencing similarly decreased AQP4 content (Fig. 4C).

Subcellular distribution of SNX27 was apparently preserved in *Kidins220*^*f/f*^ astrocytes (Supplementary Fig. S8). In addition, analysis of the early endosomal marker Rab5 revealed no substantial differences in *Kidins220*^*f/f*^ astrocytic endosomal network (Supplementary Fig. S9). Together these data suggest that the early endosomal compartment is not globally affected by Kidins220 deficiency. The retromer-independent SNX17 [54] remained constant as well, both in *Kidins220*^*f/f*^ and Kidins220 silenced astrocytes (Supplementary Fig. S10), supporting the specificity of Kidins220 deficiency on retromer regulation.

The loss of SNX27 and VPS35 in *Kidins220*^*f/f*^ deficient astrocytes, contrary to that of AQP4, was not rescued by bafilomycin A1 treatment (Supplementary Fig. S11a). In addition, *Snx27* and *Vps35* mRNA levels did not decrease in PVA tissue or cultured astrocytes from *Kidins220*^*f/f*^ mice, while *Vps35* transcripts only increased slightly in the glial cells in vitro (Supplementary Fig. S11b, c), suggesting that post-transcriptional regulation mechanisms distinct from lysosomal degradation mediate Kidins220-dependent SNX27 and VPS35 downregulation.

Kidins220 is considered as a canonical cargo for the SNX27-retromer and its type I postsynaptic density-95/discs large/zonula occludens-1 (PDZ)-binding motif or ligand (PDZ-L) has been classified as a bona fide ligand for SNX27 PDZ domain [25, 55]. Notably, AQP4 bears a PDZ-L that is fundamental for its localization at the plasma membrane in astrocytes [56, 57]. AQP4 PDZ-L is evolutionarily conserved (Fig. 4D) and highly similar to that of Kidins220 and other known SNX27 cargos (Fig. 4E), suggesting that AQP4 could be a cargo for the SNX27-retromer. In this scenario, it was feasible that SNX27 downregulation in *Kidins220*^*f/f*^ mice could lead to lysosomal processing of AQP4 in astrocytes, as it occurs with Kidins220 after SNX27 loss in tumour cells [25]. However, alignment of AQP4 PDZ-L sequence with those from consensus SNX27-retromer cargos, including Kidins220, showed an apparently important mismatch at the acidic clamp for SNX27 interaction [55] (Fig. 4E). To address whether AQP4 could be a cargo for the SNX27-retromer, we first analysed the cellular sub-localization of AQP4 and some components of this complex in cultured astrocytes from WT mice. Confocal microscopy imaging showed co-localization of AQP4 not only with SNX27 but also with VPS35 (Fig. 4F) and Kidins220 (Supplementary Fig. S12). Moreover, AQP4 was present in VPS35-immunocomplexes obtained from murine cerebral cortex and cultured primary astrocytes (Fig. 4G). We could not use SNX27 antibodies for co-immunoprecipitation experiments due to the difficulty of distinguishing the SNX27 band from that of the immunoglobulin heavy chain in the immunoblot analysis. In addition, we examined AQP4 association to the SNX27-retromer by transfecting HEK293T cells with plasmids encoding AQP4, full-length SNX27 (SNX27-FL) or a mutant lacking its PDZ-domain (SNX27-ΔPDZ), all fused to GFP. Analysis of endogenous VPS35-immunoprecipitates revealed they contained AQP4 (Fig. 4H). Co-expression of SNX27-FL is compatible with the association of AQP4 with VPS35-immunocomplexes but co-expression of SNX27-ΔPDZ disrupted this binding (Fig. 4H). Complementary experiments showed that mCherry-SNX27 was present in GFP-AQP4 immunoprecipitates (Supplementary Fig. S13). Together, these data indicate that the PDZ domain of SNX27 is necessary for the association of AQP4 with VPS35 and that AQP4 is a novel cargo for the SNX27-retromer.

### Kidins220-dependent AQP4 downregulation is mediated by SNX27

**Fig. 5:**
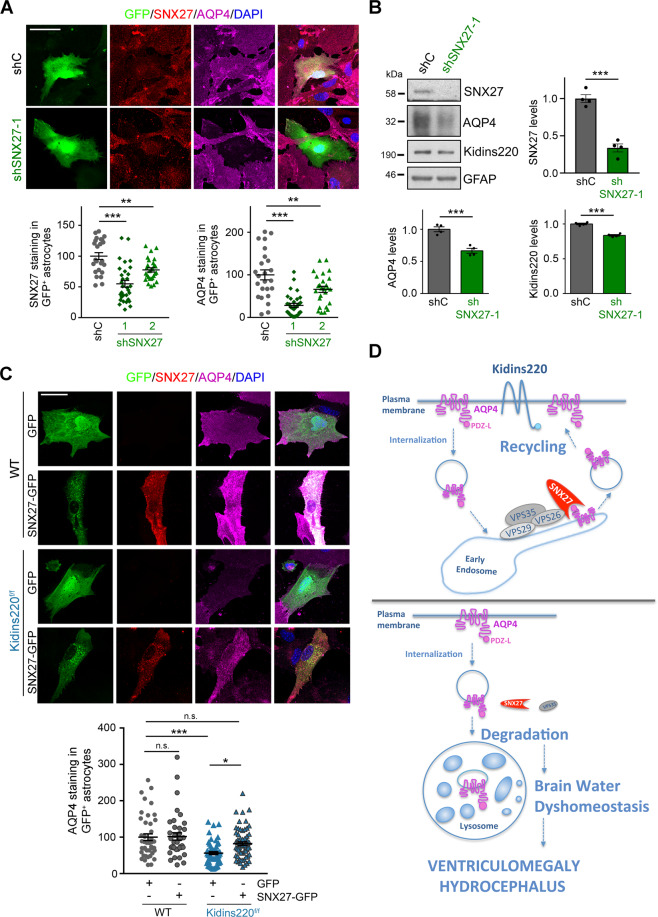
AQP4 downregulation by Kidins220 deficiency is mediated by SNX27. **A** Representative confocal microscope images of GFP (green), SNX27 (red), AQP4 (magenta) and DAPI (blue) staining of cultured astrocytes transfected with plasmids encoding GFP and shC or shSNX27 (1 and 2). Scale bar 50 μm. (Lower panels) Quantification of SNX27 and AQP4 fluorescence intensity in GFP^+^ astrocytes in each condition is shown relative to shC-transfected cells (*n* = 20–30 astrocytes per condition; *n* = 3 independent experiments); each data point represents a single astrocyte. **B** Representative SNX27, AQP4, Kidins220 and GFAP (loading control) immunoblot analyses of cortical astrocytes from WT mice transduced with shC- or shSNX27-1-encoding lentivirus. Quantification of AQP4, SNX27 and Kidins220 levels after normalization with GFAP is shown relative to shC values (*n* = 4 independent experiments). **C** Representative confocal microscopy images of GFP (green), SNX27 (red), AQP4 (magenta) and DAPI (blue) staining of cultured astrocytes from WT or *Kidins220*^*f/f*^ mice transfected with plasmids encoding GFP or SNX27-GFP. Scale bar 50 μm. Quantification of AQP4 and SNX27 fluorescence intensity in GFP^+^ astrocytes is shown relative to shC-transfected cells (40–60 astrocytes per condition; *n* = 3 independent experiments); each data point represents a single astrocyte. Quantification data are mean  ± s.e.m.; *NS* not significant; *0.01 < *P* < 0.05, **0.001 < *P* < 0.01, ***P < 0.001; by one-way ANOVA (**A**), two-tailed unpaired Student’s *t* test in (**B**) and by two-way ANOVA (**C**). **D** Scheme illustrating the role of Kidins220 in the control of AQP4 through SNX27 downregulation. In the presence of Kidins220, AQP4 levels are preserved by the interaction with the SNX27-retromer that favours AQP4 endosomal recycling to the plasma membrane. In the absence of Kidins220, SNX27 and VPS35 levels decrease drastically and AQP4 is targeted for lysosomal degradation, consequently leading to brain water dyshomeostasis and contributing to the development of ventriculomegaly and hydrocephalus.

To investigate whether SNX27 downregulation mediates the decrease of AQP4 levels caused by Kidins220 deficiency, we generated lentiviral particles for SNX27 silencing in WT cultured astrocytes (Supplementary Fig. S14a). Transduction of astrocytes with shSNX27-1 or −2 lentivirus sharply decreased AQP4 content, as assessed by immunofluorescence and quantitative immunoblot analysis (Fig. 5A, B and Supplementary Fig. S14b, c). Note that SNX27 knockdown also diminished significantly (*P* < 0.001) Kidins220 protein levels in astrocytes (Fig. 5B and Supplementary Fig. S14c), as previously observed in HeLa cells [25]. In addition, AQP4 levels were restored after lysosomal inhibition by bafilomycin treatment in SNX27-silenced astrocytes (Supplementary Fig. S14d). Conversely, forced SNX27 expression in *Kidins220*^*f/f*^ astrocytes substantially increased AQP4 expression (Fig. 5C). Of note, AQP4 colocalized with ectopic SNX27 in Rab5+ endosomes (Supplementary Fig. S15). Together our data support the notion that Kidins220 deficiency causes SNX27 and VPS35 loss that in turn mediates AQP4 lysosomal degradation and water dyshomeostasis (see scheme in Fig. 5D).

## Discussion

Here we have uncovered Kidins220-SNX27-retromer as a regulatory pathway for brain AQP4 expression and its involvement in brain ventricular enlargement and hydrocephalus. We have identified a surprising link between SNX27-retromer down-regulation and AQP4 lysosomal degradation induced by Kidins220 deficiency, and discovered that KIDINS220 and AQP4 expression is sharply decreased at the ependymal barrier in iNPH patients. We also show that hypomorphic *Kidins220*^*f/f*^ mice are a novel mouse model of hydrocephalus that display ventriculomegaly and a marked AQP4 downregulation in brain astrocytes and in the ependymal cells lining lateral ventricles, without apparent ependymocytes structural organization and ciliogenesis impairment.

The *Kidins220* hypomorphic mouse model shows various degrees of Kidins220 downregulation that positively correlate with ventriculomegaly penetrance. The decrease in the number of *Kidins220*^*f/f*^ mice at weaning might be attributed to embryonic or perinatal hydrocephalus-associated death of mice with high downregulation of Kidins220. Supporting this hypothesis, constitutive *Kidins220*^*−/−*^ mice die at birth and their brains present a pronounced ventricular enlargement [43]. The majority of *Kidins220*^*f/f*^ born mice reached adulthood without showing apparent external evidence of overt hydrocephalus, suggesting that ventricle enlargement progresses slowly in these animals. While the brain of *Kidins220*^*−/−*^ embryos presents a pronounced enlargement of LVs, TV and FV [43], 2-month-old *Kidins220* ^*f/f*^ animals only show enlargement of the LVs and TV. These results suggest an incipient nature of hydrocephalus in 2-month-old *Kidins220*^*f/f*^, which might develop progressively with aging. Ventriculomegaly in these mice resembles that observed in *Aqp4*^*−/−*^ animals, which show sporadic progressive obstructive hydrocephalus with LVs and TV enlargement and aqueduct stenosis, without severe changes in FV volume and mild ependymal disorganization [14, 58]. In contrast, ependymal-conditional *Aqp4*^*−/−*^ mice reveal no abnormalities in the ependymal lining and do not present changes in brain ventricular volume or increased brain water content, at least at the age studied [18]. A possibility is that ependymal-conditional *Aqp4*^*−/−*^ mice could develop ventricular enlargement during aging, a study that is indeed missing. Alternatively, these data could suggest that ventriculomegaly in *Kidins220*^*f/f*^ mice might rely not only on AQP4 downregulation but also on changes in additional molecules.

Constitutive *Snx27*^*−/−*^ mice develop severe hydrocephalus soon after birth, showing ependymal deciliation and differentiation defects with complete penetrance [24]. These mice could show a more severe phenotype than *Kidins220*^*f/f*^ animals because the latter do not completely lack SNX27, thus sparing its cargos from complete lysosomal degradation.

A very recent publication, that does not study AQP4, describes that young postnatal *Vps35* conditional KO mice develop neonatal hydrocephalus, similar to that of *Snx27*^*−/−*^ mice, with deficient ependymal cells differentiation and ciliogenesis [59]. As in *Kidins220*^*f/f*^ animals, S100β is downregulated in the ependymal barrier of *Vps35*-deficient mice. Locally activated microglia seems to be responsible for ependymal cells dyshomeostasis provoked by VPS35 loss, as pathology diminishes following microglia depletion [59]. A similar environment, with activated microglia exerting cell non-autonomous effects might be found in *Kidins220*^*f/f*^ ependymal barrier where VPS35 levels are also severely decreased. Alternatively, as ependymal cells precursors are S100β negative and mature ependymal cells are S100β positive, the lack of S100β staining might indicate changes in the different cell populations at the ependymal barrier in Kidins220 deficient animals. Future studies to analyse in detail the different cellular subtypes that constitute the ventricular and subventricular zones (i.e. number of precursors and their proliferation) or the presence of activated microglia will be of high interest. Cell non-autonomous effects might also modify the nature of astrocytes in *Kidins220*^*f/f*^ brain that may retain these altered features when cultured in vitro. This scenario would explain why VPS35 levels are decreased in cultured *Kidins220*^*f/f*^ astrocytes while they remain unaltered following Kidins220 silencing in cultured WT astrocytes. These results are in striking contrast to those obtained for SNX27, which diminishes both in *Kidins220*^*f/f*^ and Kidins220-silenced astrocytes, indicating a cell-autonomous and direct role of Kidins220 on SNX27 regulation but not on VPS35.

Future studies will determine whether the genetic inactivation of *Snx27* or *Vps35* leads to AQP4 depletion and if restoration of SNX27-retromer activity in vivo can be of therapeutic interest to stabilise AQP4 levels and rescue ventriculomegaly in *Kidins220*-deficient mice or in other hydrocephalus or neurological disease models that may concur with AQP4 deficiency. We propose that AQP4 downregulation is critical for ventricular enlargement in *Kidins220*^*f/f*^ animals, but we cannot rule out that other proteins deregulated in these mice might also contribute to this phenotype. This could be the case of transient receptor potential vanilloid 4 (TRPV4), a cation channel upregulated in *Kidins220*^*−/−*^ astrocytes [60] that together with AQP4 is essential for water cell-volume control in these cells [52].

Kidins220 trafficking from the endosome to the plasma membrane or to the lysosome is regulated in HeLa cells by the SNX27-retromer [25], a complex responsible for the endosomal recycling of multiple cargos to the cellular surface [26]. Although regulation in the reverse direction (of SNX27-retromer levels by Kidins220) was unexpected, here we find that Kidins220 deficiency downregulates SNX27 in vivo and in vitro, and VPS35 in vivo, likely through protein stabilization, as mRNA levels for both molecules are not substantially decreased. The possible role of Kidins220 on the regulation of SNX27-retromer stability is extremely attractive and deserves further investigation.

Globally, our results point to Kidins220 as a critical regulator of SNX27-retromer dependent endosomal sorting of multiple cargos. Accordingly, a genome-wide analysis performed in *Caenorhabditis elegans* identified Kidins220 as a strong candidate regulator of endocytosis [61]. Kidins220 and SNX27, therefore, regulate one another in an intriguing reciprocal ‘feedback loop’ that may result in a fine-tuning of endocytic recycling versus lysosomal degradation of various SNX27-retromer cargos. Whether Kidins220 loss could affect AQP4 directly is an interesting issue difficult to study since Kidins220 deficiency decreases both SNX27 and AQP4. Our studies using SNX27 silencing and rendering AQP4 downregulation, suggest that AQP4 downregulation in Kidins220-deficient cells and mice is mediated by SNX27 protein loss, but we cannot exclude that an additional, direct regulation of Kidins220 on AQP4 (SNX27-independent) may also exist.

Here we have identified AQP4 as a novel SNX27-retromer cargo. AQP4 C-terminal amino acids constitute a canonical type I PDZ-L, but differ slightly with those defined as selective for SNX27 PDZ-binding bearing an acidic clamp between P-3 and P-5 [55]. Similar to other SNX27 cargos, such as the typical cargo GLUT1, or PTEN [62] and OTULIN [63], AQP4 PDZ-L does not contain acidic residues at both P-3 and P-5, suggesting that selectivity and affinity of SNX27 for its cargos may rely not only on their C-terminal peptide but also on their global context.

*KIDINS220* heterozygous nonsense variants have been recently associated with SINO syndrome, a novel rare autosomal disease characterized by spastic paraplegia, intellectual disability, nystagmus and obesity in children [40]. Similar to *Kidins220*^*f/f*^ mice, the brain LVs and TV of these children are enlarged but not the FV [40]. Moreover, homozygous *KIDINS220* loss-of-function variants have been detected in foetuses that also present cerebral ventriculomegaly [41]. We anticipate that truncated Kidins220 forms lacking the PDZ-L, as that identified in SINO syndrome [40], will not interact with the SNX27-retromer complex. Recycling to the cell surface of Kidins220 variants devoid of PDZ-L would therefore be hampered and these variants would be mostly targeted for lysosomal degradation. Accordingly, this type of variant would not only greatly impair Kidins220 function at the cell surface and other intracellular compartments, but also drop its steady-state levels by increasing its degradation rate. Moreover, as the interaction of Kidins220 PDZ-L with SNX27 could be key for maintaining SNX27-retromer stability and levels, the lack of Kidins220 PDZ-L binding could destabilize SNX27 and therefore decrease its half-life. The global recycling of SNX27 cargos (like AQP4) to the cell surface would be consequently diminished, resulting in their increased lysosomal targeting and degradation. SINO syndrome, hydrocephalus and ventriculomegaly could thus be all retromer-related disorders.

The novel missense variants identified in *KIDINS220* gene in schizophrenic patients [37–39] could be also associated with cerebral ventricular enlargement in these individuals given it is a frequent feature in this psychiatric disorder [5, 6], a possibility that should be examined. Although it is currently unknown whether KIDINS220 variants in SINO and schizophrenic patients could lead to similar defects in SNX27-retromer and AQP4 expression and brain water dyshomeostasis as found in *Kidins220* deficient mice, our data point at *Kidins220*^*f/f*^ mice as an attractive model to study SINO syndrome, Kidins220-linked schizophrenia and other neuropathologies accompanied by ventriculomegaly such as foetal/perinatal to adulthood onset forms of hydrocephalus.

The complex etiopathology of ventriculomegaly and hydrocephalus and the limited knowledge of the molecular mechanisms involved hinder the diagnostic, prognostic and therapeutic strategies for patients. There is an urgent need of appropriate tools for differential diagnosis and biomarkers for specific hydrocephalus types or different ventriculomegaly-associated diseases, especially in adults. The most common procedure to treat the large ventricular accumulation of CSF, the implantation of a ventricular shunt, saves the life of many patients but the failure rate of this procedure is high and its outcome is not always positive. AQP4 downregulation had been shown at perivascular locations in iNPH patients [22, 23] and here we show that its expression and that of KIDINS220 (but not S100β) are substantially decreased at the ventricular ependymal lining, suggesting that loss of these proteins at the ependyma might also contribute to adult chronic hydrocephalus. Indeed, AQP4 deficiency in this region might account for the reported enhancement in transependymal CSF flow in iNPH patients [64] that also present glymphatic (glia-lymphatic) circulation system for brain waste clearance impairments [64, 65]. Since the glymphatic CSF circulation is controlled by AQP4 and is altered in iNPH and AD [11], our results prompt to investigate the participation of KIDINS220 and SNX27-retromer in the function of this system, and in the dynamics and turnover of AQP4 and other cargos in iNPH and different hydrocephalus types, AD, and other neurodegenerative and neuropsychiatric disorders that concur with ventriculomegaly. Novel strategies designed to potentiate AQP4 levels in ventricular ependymal cells and astrocytes, including the use of viral vectors, molecules or compounds that enhance Kidins220 and/or SNX27-retromer expression or stabilization and therefore AQP4 recycling to the plasma membrane, could be promising therapeutic approaches to prevent or revert ventricular enlargement caused by AQP4 increased turnover.

## Supplementary information

SUPPLEMENTAL FIGURES AND TABLES

SUPPLEMENTAL MATERIALS AND METHODS
