# Supplementary material for: Kidins220 deficiency causes ventriculomegaly via SNX27-retromer-dependent AQP4 degradation
Source: Mol Psychiatry. 2021 May 17;26(11):6411–26. doi: 10.1038/s41380-021-01127-9 (PMC8760065; doi:10.1038/s41380-021-01127-9)
Supplement: Supplementary file 1 — SUPPLEMENTAL FIGURES AND TABLES [file 41380_2021_1127_MOESM1_ESM.pdf]

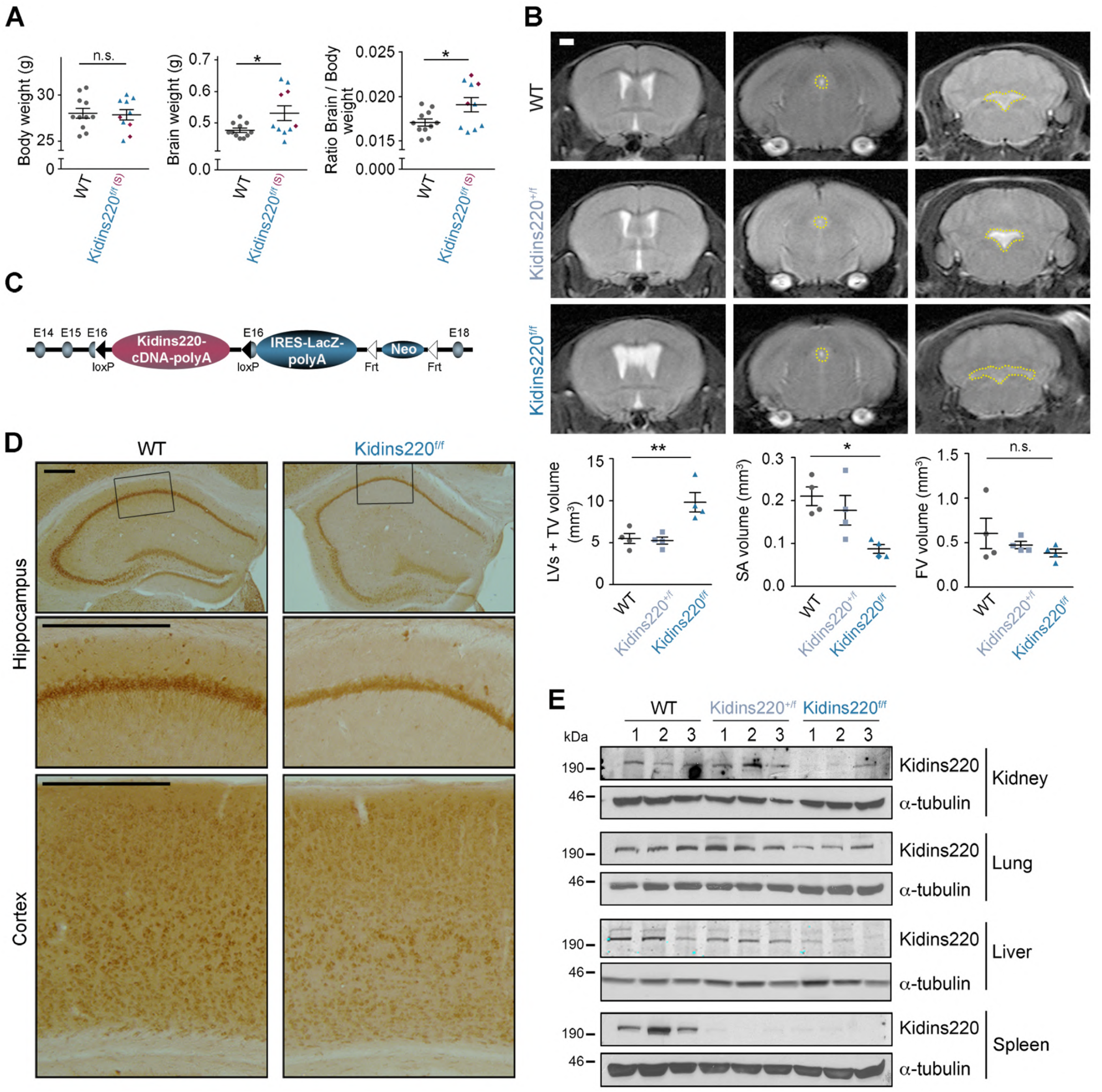

Supplementary Figure 1. Phenotypic features of *Kidins220*$^{f/f}$ mice. a Representation of body (left panel) and brain (middle) weight and their ratio (right panel) in 11 WT, 7 *Kidins220*$^{f/f}$ (triangles), and 3 *Kidins220*$^{f/f(S)}$ (diamonds) 2-months-old mice. **b** Representative coronal T2-W MRI images showing lateral and third ventricles (LVs+TV; left panels), aqueduct of Sylvius (SA; central panels, white square), and fourth ventricle (FV; right panels) of 2-month-old WT, *Kidins220*$^{f/+}$and *Kidins220*$^{f/f}$ female mice. Scale bar, 1 mm. Quantification of ventricular and aqueduct volume (mm^3) in 4 WT, 4 *Kidins220*$^{f/+}$ and 4 *Kidins220*$^{f/f}$ (triangles) female mice. **c** Scheme of genetic manipulation to generate *Kidins220*$^{f/f}$ mice. *Kidins220* cDNA flanked by two loxP sequences was introduced in exon 16 of *Kidins220* mouse gene (for further details see Cesca et al. Cell Death Differ. 2012; 19:194-208). **d** Representative images of Kidins220 DAB immunostaining of the hippocampus (with zoom-boxed CA1 region) and the cortex from WT and *Kidins220*$^{f/f}$ mice. Scale bar, 50 µm. **e** Kidins220 and α-tubulin (loading control) immunoblots of kidney, lung, liver and spleen lysates from WT, *Kidins220*$^{f/+}$, and *Kidins220*$^{f/f}$ mice; each lane represents one mouse. (a,b) Data are mean ± s.e.m. and each data point represents an individual mouse. n.s, not significant; *0.01<P<0.05, **0.001<P<0.01; by two-tailed unpaired Student's t test (**a**), and one-way ANOVA (**b**).

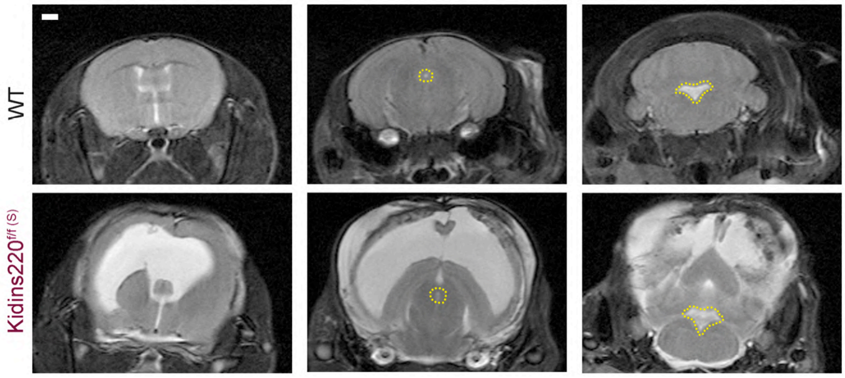

Supplementary Figure 2. MRI of a *Kidins220*$^{f/f}$(s) mouse with highly severe hydrocephalus phenotype. *In vivo* T2-weighted (T2-W) MRI coronal images showing lateral and third ventricles (left panels), aqueduct of Sylvius (central panels) and fourth ventricle (right panels) of 2-month-old WT, and *Kidins220*$^{f/f}$*(S)* male mice. Scale bar, 1 mm.

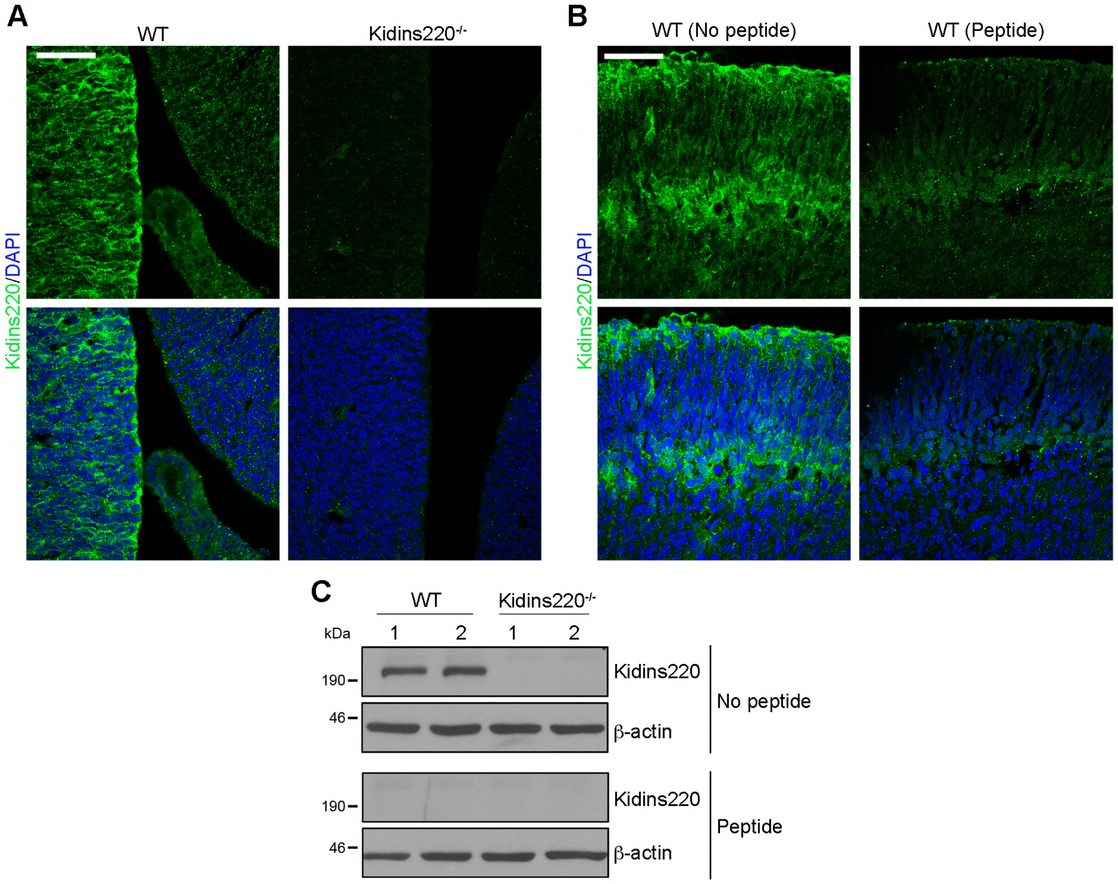

Supplementary Figure 3. Validation of Kidins220 antibody specificity. a Representative confocal microscopy images showing Kidins220 (green) immunostaining and DAPI nuclear labelling (blue) of E16 embryonic brain from WT and constitutive *Kidins220*$^{-/-}$ mice. Scale bar, 50 µm. **b** Immunostaining of brain cortex sections from 2-month-old WT mice with Kidins220 antibody neutralized (Peptide) or not (No peptide) with the immunizing peptide. Scale bar, 50 µm. **c** Representative Kidins220 and β-actin (loading control) immunoblots of E16 embryonic brain lysates from WT and *Kidins220*$^{-/-}$ mice using Kidins220 antibody before or after peptide-neutralization; each lane represents one mouse.

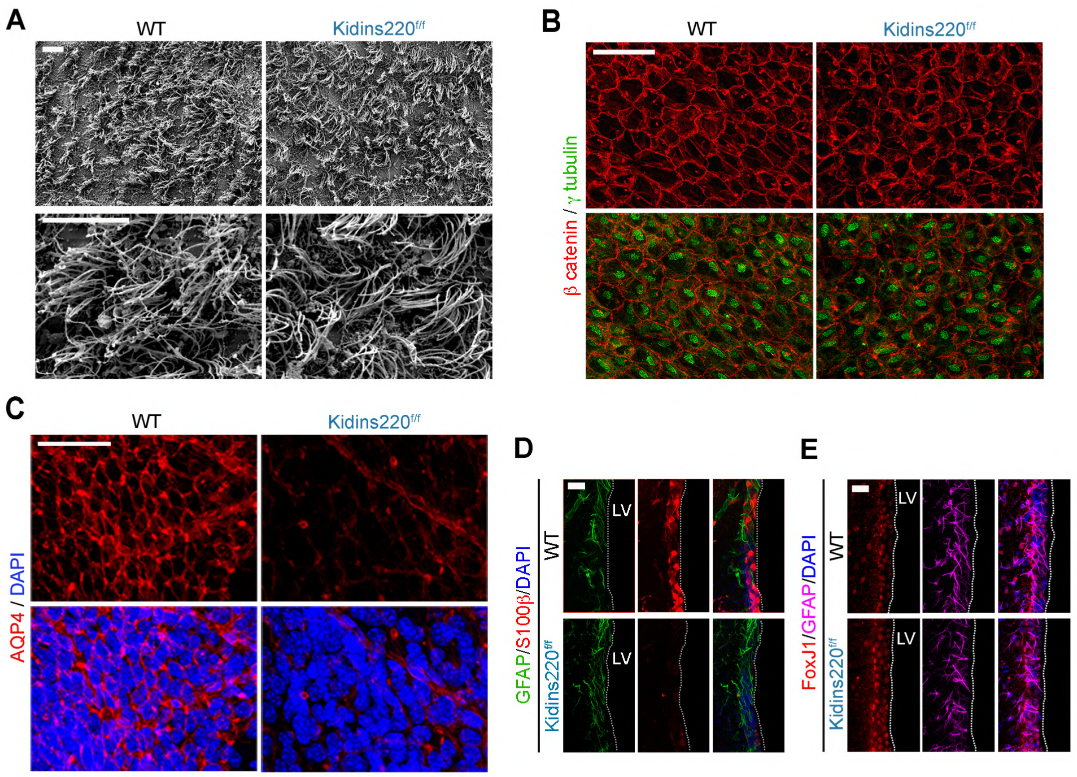

Supplementary Figure 4. Ependymocytes of *Kidins220$^{f/f}$* mice present normal cilia and planar polarity organization, and ependymal barrier contains unaltered GFAP and FoxJ1 staining. a Representative scanning electron microscopy images of ependymal cilia from lateral ventricles of 2-month-old WT and *Kidins220$^{f/f}$* mice. Scale bar, 10 µm. **b** Representative confocal microscopy images of β-catenin (red) and γ-tubulin (green) immunostaining of whole-mount preparations showing the ependymal layer adherent junctions and the ependymal basal body, respectively, at the ventricle surface of of 2-month-old WT and *Kidins220$^{f/f}$* mice. Scale bar, 50 µm. **c** Representative AQP4 (red) and DAPI (blue) staining of whole-mount preparations of 2-month-old WT and *Kidins220$^{f/f}$* mice lateral ventricles. Scale bar, 50 µm. **(d, e)** Representative confocal microscopy images of GFAP (green), S100β (red) in (**d**) or FoxJ1 (red), GFAP (magenta) in (**e**) and DAPI (blue) staining of the ependymal barrier from lateral ventricles (LV) of WT and *Kidins220$^{f/f}$* mice. Scale bar, 25 µm.

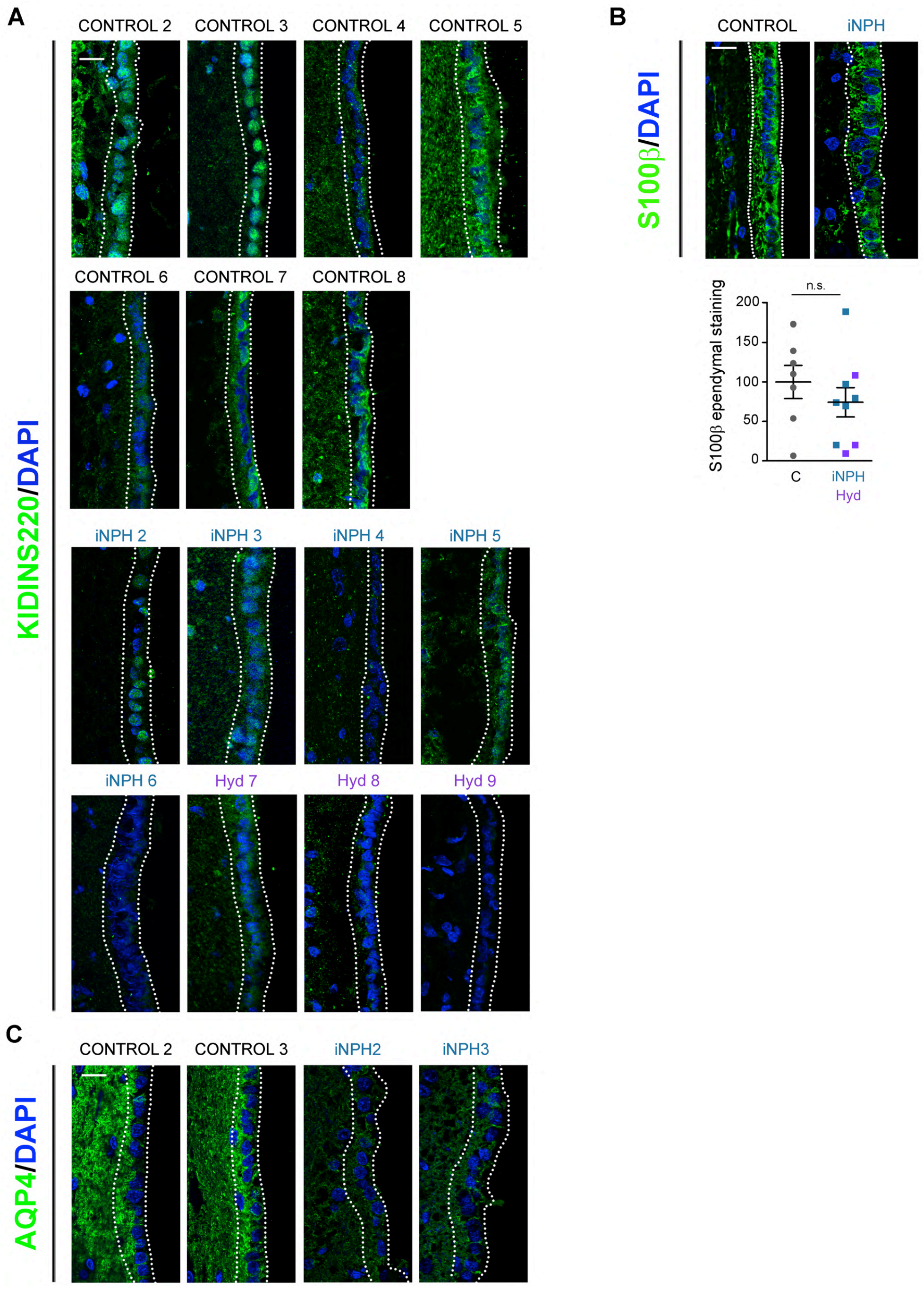

Supplementary Figure 5. KIDINS220 and AQP4, but not S100β, are downregulated in the ependymal barrier of hydrocephalic patients. Representative confocal microscopy images of KIDINS220 (**a**), S100β (**b**) and AQP4 (**c**) immunostaining (green) and DAPI nuclear labelling (blue) at the ependymal barrier in postmortem necropsies from control subjects, idiopathic normal pressure hydrocephalus patients (iNPH) and non-normotensive hydrocephalic patients (Hyd). Dotted line delimits the ROI analysed in each image. Scale bar, 30 µm. Quantification of S100β immunostaining at the ependymal barrier in (**b**) of hydrocephalic patients (iNPH n=6 and Hyd n=3) and control subjects (n=7). Each data point denotes an individual subject. Non-normotensive hydrocephalic patients (Hyd) are depicted in purple. For quantifications, 9 randomly selected ROIs per section where used; data are mean ± s.e.m. n.s, not significant by two-tailed unpaired Student's *t* test.

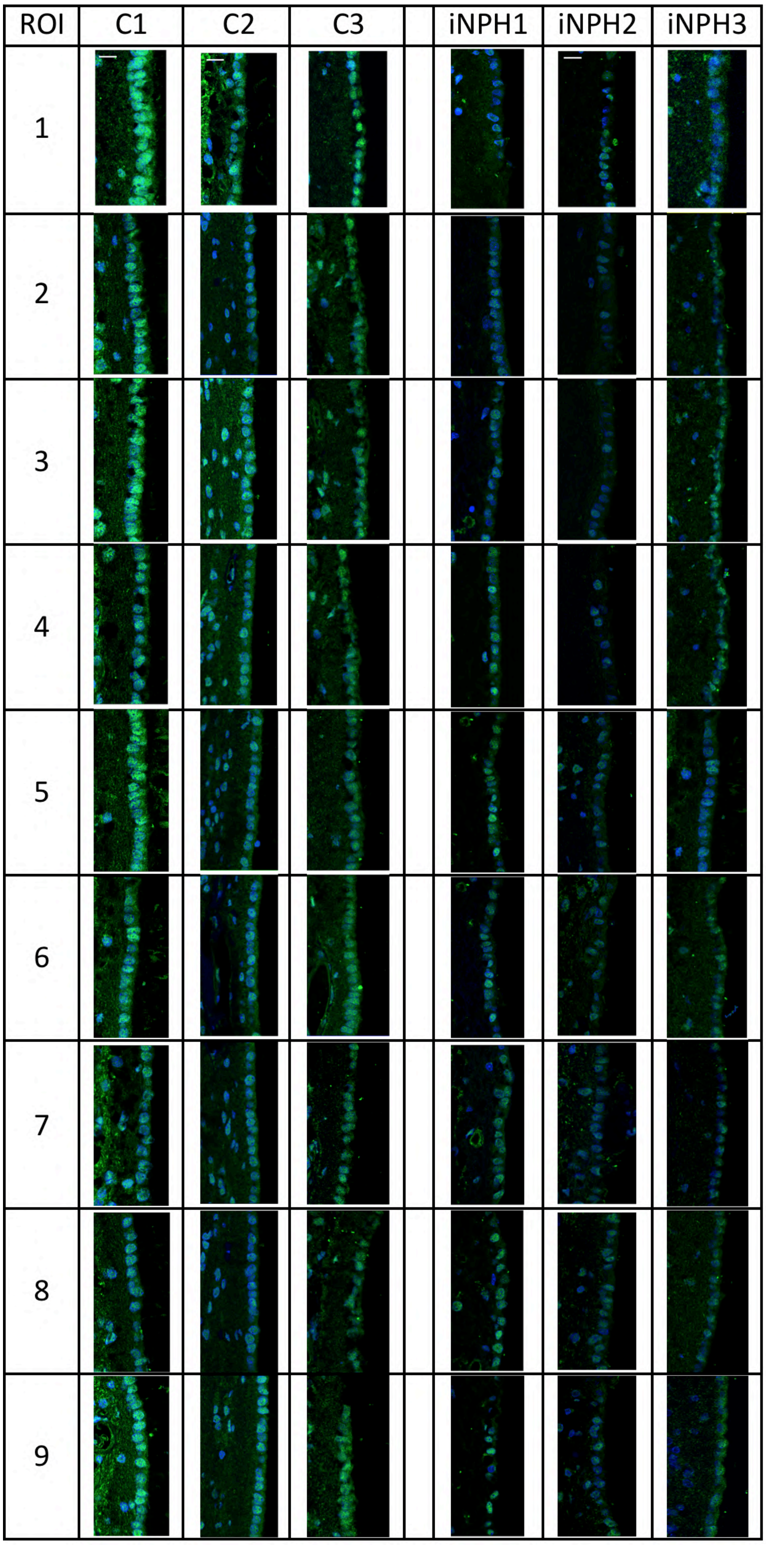

Supplementary Figure 6: **ROIs of KIDINS220 immunostaining in human control and iNPH samples.** Confocal microscopy images of KIDINS220 (green) and DAPI (blue) staining from 9 different ROIs quantified from 3 representative control subjects (C1, C2, C3) and 3 iNPH patients (iNPH1, iNPH2, iNPH3) are shown.

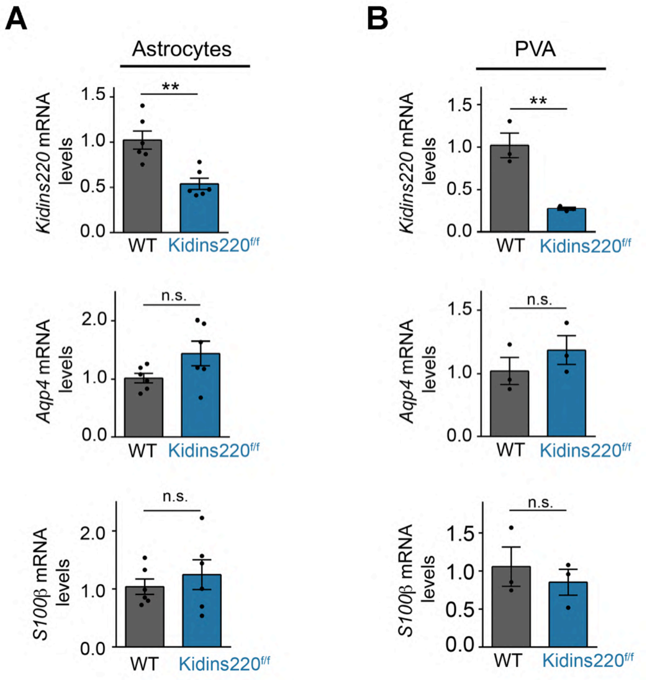

Supplementary Figure 7. Kidins220 deficiency does not alter *Aqp4* and *S100β* gene expression. RT-qPCR analysis of *Kidins220*, *Aqp4* and *S100β* mRNA levels in (a) primary cultured astrocytes (n=6 independent cultures per genotype) or (**b**) the periventricular area (PVA) (n=3 animals per genotype) obtained from 2-month-old WT and *Kidins220$^{f/f}$* mice. Data are shown relative to WT mice as mean ± s.e.m.; each data point represents an individual mouse. n.s., not significant; **0.001<*P*<0.01; by two-tailed unpaired Student's *t* test.

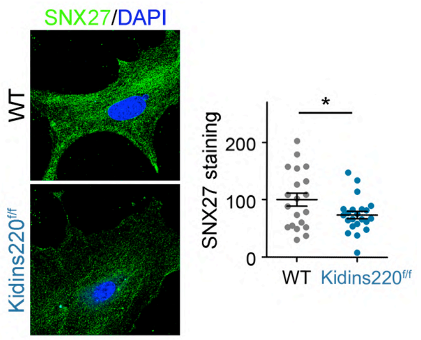

Supplementary Figure 8. SNX27 decreases in *Kidins220$^{f/f}$* astrocytes preserving its subcellular distribution. Representative confocal microscope images of SNX27 (green) and DAPI (blue) staining of cultured astrocytes from WT or *Kidins220$^{f/f}$* mice and quantification (right panel) of SNX27 fluorescence intensity; data are mean ± s.e.m. and each data point represents a single astrocyte (n=10-20 astrocytes per condition; n=3 independent experiments). *P<0.05 by two-tailed unpaired Student's *t* test.

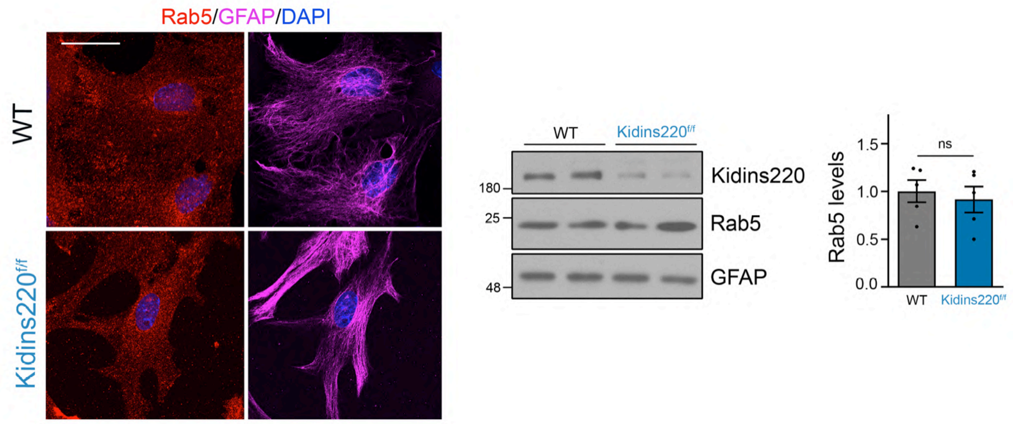

Supplementary Figure 9. *Kidins220$^{f/f}$* astrocytes show normal Rab5 early endosomal compartment and levels. Representative confocal microscopy images of Rab5 (red) and GFAP (magenta) staining of primary cortical astrocytes dissected from WT (n=3) and *Kidins220$^{f/f}$* (n=3) mice. Nuclei are stained with DAPI. Scale bar: 25 µm. Kidins220, Rab5 and GFAP (loading control) immunoblot analyses of WT and *Kidins220$^{f/f}$* cortical astrocytes; each lane represents a culture obtained from one animal (n=5 animals per genotype). Graphs represent the quantification of Rab5 after normalization with GFAP. Quantification data are shown as mean ± s.e.m.; n.s, not significant by two-tailed unpaired Student`s t test.

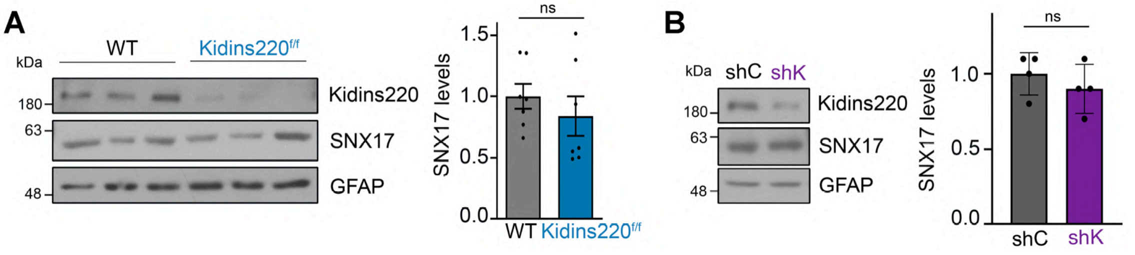

Supplementary Figure 10. Kidins220 deficiency in astrocytes does not alter the levels of the retromer-independent SNX17. Kidins220, SNX17 and GFAP (loading control) immunoblot analyses of WT and *Kidins220$^{f/f}$* cortical astrocytes (left panels) and cultured cortical astrocytes obtained from WT mice transduced with lentivirus encoding shC or shK (right panels). Graphs represent the quantification of SNX17 after normalization with GFAP. Quantification data are shown as mean ± s.e.m.; n.s, not significant; by two-tailed unpaired Student`s t test.

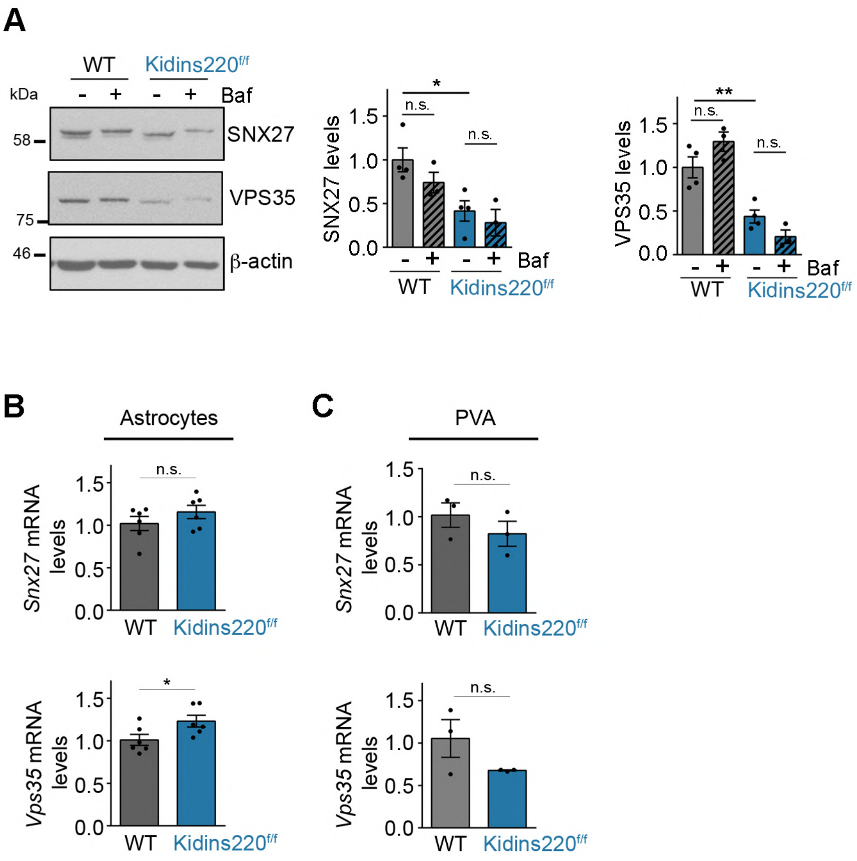

Supplementary Figure 11. Kidins220 deficiency downregulates SNX27 and VPS35 by post-transcriptional mechanisms independent of lysosomal degradation. a Representative SNX27, VPS35 and β-actin (loading control) immunoblot analysis of cultured cortical astrocytes obtained from WT and *Kidins220$^{f/f}$* mice treated with vehicle (-) or bafilomycin A1 (Baf, +). Quantification of SNX27 and VPS35 levels after normalization with β-actin (n=4 independent experiments). **b,c** RT-qPCR analysis of *Snx27* and *Vps35* mRNA levels in (**b**) primary cultured astrocytes (n=6 independent cultures per genotype) or (**c**) the periventricular area (PVA) (n=3 animals per genotype) obtained from 2-month-old WT and *Kidins220$^{f/f}$* mice. Data are shown relative to WT mice as mean ± s.e.m.; each data point represents an individual mouse. n.s, not significant; *$0.01<P<0.05$; **$0.001<P<0.01$; by two-way ANOVA (**a**) or by two-tailed unpaired Student's *t* test (**b, c**).

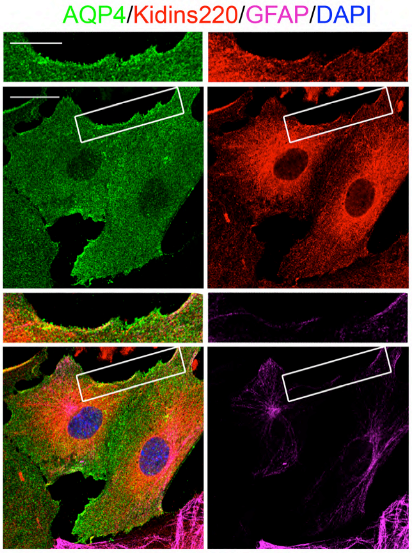

Supplementary Figure 12: Kidins220 and AQP4 colocalization at the plasma membrane of cultured astrocytes. Representative confocal microscopy images of AQP4 (green), Kidins220 (red), GFAP (magenta) and DAPI (blue) staining of primary cortical astrocytes dissected from WT (n=3) mice. Zoom images from boxed regions are also shown. Scale bars, 20 and 10 (magnifications) µm.

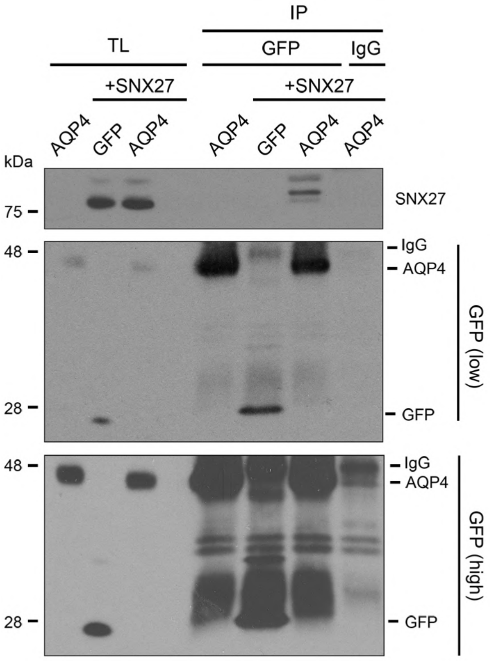

Supplementary Figure 13. SNX27 coimmunoprecipitates with AQP4 in transfected HEK293T cells. Protein extracts from HEK293T cells transfected with GFP or GFP-AQP4, and mCherry-SNX27 (SNX27) were immunoprecipitated with an antibody against GFP and the indicated proteins were detected in GFP-immunocomplexes and in the total lysates by immunoblot. Low and high exposures of the immunoblots are depicted in order to allow proper analysis of all the existing bands (n=3 independent experiments).

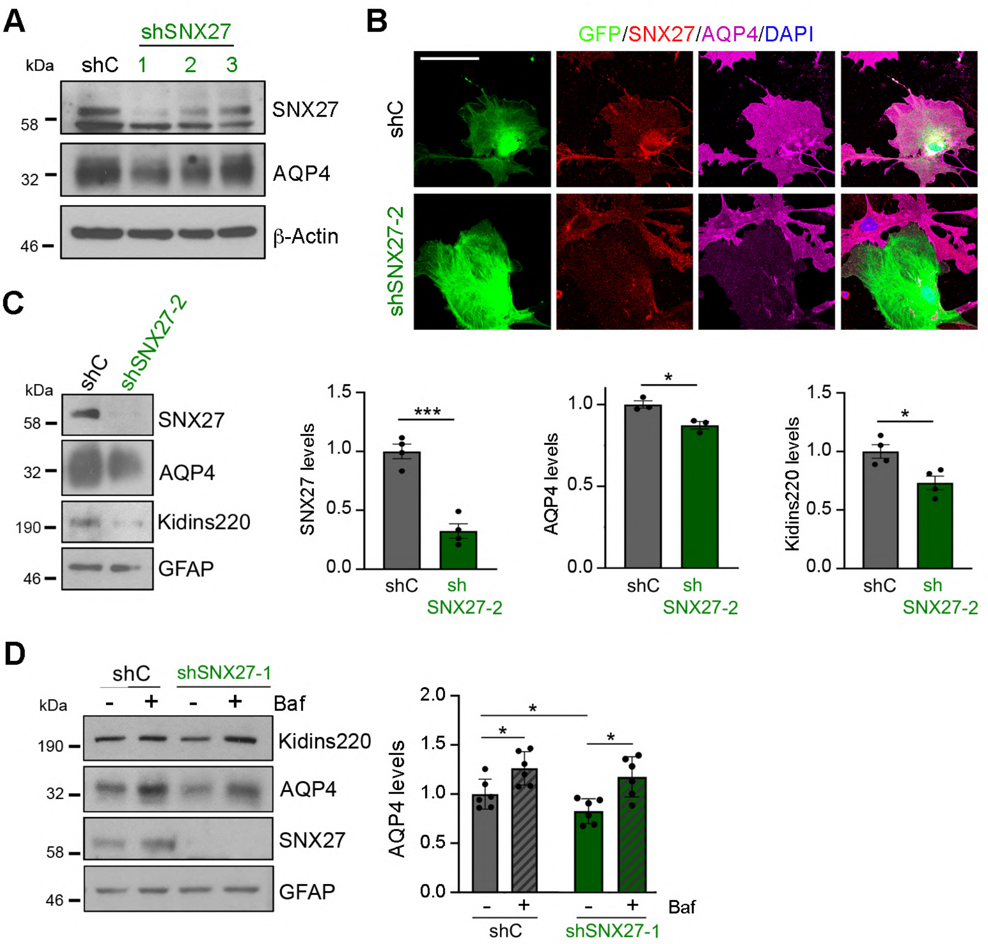

Supplementary Figure 14. SNX27 silencing decreases astrocytic AQP4 levels by lysosomal degradation. a SNX27, AQP4 and β-actin (loading control) immunoblot analysis of mouse cortical astrocytes transduced with lentiviral particles encoding shC or 3 independent *SNX27*-specific shRNA sequences (shSNX27-1, -2 and -3). **b** Representative confocal microscope images of GFP (green), SNX27 (red), AQP4 (magenta) and DAPI (blue) staining of cultured astrocytes transfected with plasmids encoding GFP and shC or shSNX27-2 (n=3 independent experiments). Scale bar, 50 µm. **c** Representative SNX27, AQP4, Kidins220, and GFAP (loading control) immunoblot analyses of cortical astrocytes from WT mice transduced with shC or shSNX27-2 encoding lentivirus. Quantification of AQP4, SNX27, and Kidins220 levels after normalization with GFAP is shown relative to shC values (n=4 independent experiments). **d** Representative Kidins220, AQP4, SNX27, and GFAP (loading control) immunoblot analysis of cultured cortical astrocytes from WT mice incubated with vehicle (-) or bafilomycin A1 (Baf, +). Quantification of AQP4 levels after normalization with GFAP signal (n=6 independent experiments) and relative to shC-transduced cells is shown. Quantification data are shown as mean ± s.e.m.; $*0.01<P<0.05$, $***P<0.001$; by two-tailed unpaired Student's *t* test in (**c**) and two-way ANOVA in (**e**).

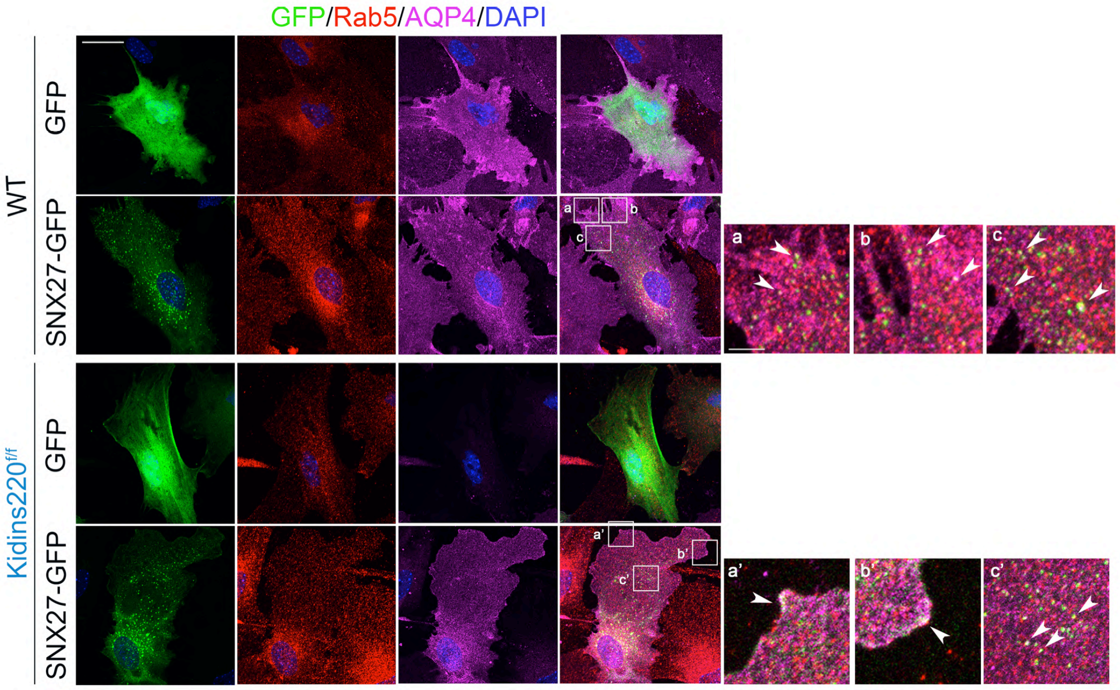

Supplementary Figure 15. Colocalization of SNX27 and AQP4 in Rab5 positive vesicles in astrocytes. Representative confocal microscopy images of GFP or GFP-SNX27 (green), Rab5 (red), AQP4 (magenta), and DAPI (blue) staining of transfected cultured astrocytes from WT or *Kidins220*$^{f/f}$ mice (n=3 independent experiments). Zoom images from boxed regions are also depicted. Arrowheads point SNX27-GFP, Rab5 and AQP4 colocalization. Scale bars, 25 µm and zoom 10 µm.

Supplementary Table 1. Details of the post-mortem brain samples from human control individuals and hydrocephalic patients. Age, gender and post-mortem interval (PMI) in hours, of control subjects (C), idiopathic normal pressure hydrocephalus (iNPH) and non-normotensive hydrocephalic patients (Hyd).

Sample ID	Age	Gender	PMI
C-1	53	M	7
C-2	56	M	3
C-3	60	F	11
C-4	83	F	4
C-5	86	M	7
C-6	83	F	7.5
C-7	93	F	5.5
C-8	83	F	7.5
iNPH-1	70	M	8-11
iNPH-2	96	M	5-7
iNPH-3	56	M	10
iNPH-4	85	F	9
iNPH-5	73	M	6.5
iNPH-6	91	F	15.3
Hyd-7	74	F	8.5
Hyd-8	87	F	8.75
Hyd-9	86	F	5.5

Supplementary Table 2. Details of the antibodies used.

Antibody	Supplier	Catalog number	Dilution
AQP4	Sigma-Aldrich	A5971	WB 1:500<br>IMF 1:500*-1:100**
β-actin	Sigma-Aldrich	A5441	WB 1:5000
β-catenin	Cell Signaling Technology	#9587	IMF 1:100*
FoxJ1	ThermoFisher Scientific	14-9965-82	IMF 1:300*
GFAP	Abcam	ab53554	IMF 1:1000*
GFAP	ThermoFisher Scientific	MA5-12023	WB 1:2000<br>IMF 1:1000*
GFP	ThermoFisher Scientific	A6455	IMF 1:500*
Kidins220	Abcam	ab34790	WB 1:1000<br>IHC 1:500*
Kidins220	Abcam	ab20211	IMF 1:100*
Kidins220	T. Iglesias[1]	-	WB 1:1000<br>IMF 1:500*<br>1:100**
Rab5	Synaptic Systems	108011	WB 1:1000<br>IMF 1:500*
S100β	Sigma-Aldrich	S2532	IMF 1:500*<br>1:50**
SNX17	Proteintech	10275-1-AP	WB 1:1000
SNX27	Abcam	ab77799	WB 1:500<br>IMF 1:500*
γ-tubulin	Sigma-Aldrich	T6557	IMF 1:300*
α-tubulin	Sigma-Aldrich	T9026	WB 1:10000
VPS35	Abcam	ab10099	WB 1:1000<br>IMF 1:500*
HRP anti-Rabbit	Santa Cruz Biotechnology	sc-2004	WB 1:5000

HRP anti-Mouse	Santa Cruz Biotechnology	sc-2005	WB 1:5000
DONKEY anti GOAT 488	Thermo Fisher Scientific	A11055	IMF 1:500*
DONKEY anti GOAT 546	Thermo Fisher Scientific	A11056	IMF 1:500*
DONKEY anti MOUSE 488	Thermo Fisher Scientific	A21202	IMF 1:500*/**
DONKEY anti MOUSE 546	Thermo Fisher Scientific	A10036	IMF 1:500*
DONKEY anti MOUSE 647	Thermo Fisher Scientific	A31571	IMF 1:500
DONKEY anti RABBIT 488	Thermo Fisher Scientific	A21206	IMF 1:500*/**
DONKEY anti RABBIT 555	Thermo Fisher Scientific	A31572	IMF 1:500*
DONKEY anti RABBIT 647	Thermo Fisher Scientific	A31573	IMF 1:500*
GOAT anti MOUSE 488	Thermo Fisher Scientific	A11029	IMF 1:500*
GOAT anti MOUSE 546	Thermo Fisher Scientific	A11030	IMF 1:500*
GOAT anti MOUSE 647	Thermo Fisher Scientific	A21236	IMF 1:500*
GOAT anti RABBIT 488	Thermo Fisher Scientific	A11034	IMF 1:500*
GOAT anti RABBIT 546	Thermo Fisher Scientific	A11035	IMF 1:500*
GOAT anti RABBIT 647	Thermo Fisher Scientific	A21245	IMF 1:500*

*Cultured astrocytes and mouse brain samples **Human brain samples

[1]Lopez-Menendez, C. et al. Excitotoxic targeting of Kidins220 to the Golgi apparatus precedes calpain cleavage of Rap1-activation complexes. Cell Death Dis 10, 535 (2019).

Supplementary Table 3. List of oligonucleotides used for quantitative real-time PCR and generation of shRNA silencing vectors.

**RT-qPCR Target gene**	**Sequence 5´-3´**
Aqp4	Forward: TTGCTTTGGACTCAGCATTG Reverse: GGGAGGTGTGACCAGGTAGA
Snx27	Forward: GCCATACTGCACCAAAACCT Reverse: TCACCAATGACTCGGATTG
S100β	Forward: GGTTGCCCTCATTGATGTCT Reverse: GTCCAGCGTCTCCATCACTT
Vps35	Forward: AACACAGAAATCGTCTCTCAGG Reverse: GCATGGACCCCTCTCTACAA
Kidins220	Forward: CCACGGCACAGTAATCTGAG Reverse: TGCACCTTATCAGTAAGCTTTGA
Gapdh	Forward: TGCGACTTCAACAGCAACTC Reverse: CTTGCTCAGTGTCCTTGCTG
**shRNA name**	**Sequence 5´-3´**
shControl	Forward: TCAACAAGATGAAGAGCACCAATTCAAGAGATTGGTGCTCTTCATCTTGTTGTTTTTC<br>Reverse: TCGAGAAAAAACAACAAGATGAAGAGCACCAATCTCTTGAATTGGTGCTCTTCATCTTGTTG A
shSNX27-1	Forward: GTGCACTGTTCAAGCATATATAGAATTCTATATATGCTTGAACAGTGCATTTTTC<br>Reverse: TCGAGAAAAATGCACTGTTCAAGCATATATAGAATTCTATATATGCTTGAACAGTGCAC
shSNX27-2	Forward: GAGCTGGAGAACCAGGTAATAGGAATTCCTATTACCTGGTTCTCCAGCTTTTTTC<br>Reverse: TCGAGAAAAAAGCTGGAGAACCAGGTAATAGGAATTCCTATTACCTGGTTCTCCAGCTC
shSNX27-3	Forward: GCCTAATGAATTTCCTCATAAAGAATTCTTTATGAGGAAATTCATTAGGTTTTTC<br>Reverse: TCGAGAAAAACCTAATGAATTTCCTCATAAAGAATTCTTTATGAGGAAATTCATTAGGC
shKidins220	Forward: GTGTCATAGCTCGGATGTCCAGAATTCTGGACATCCGAGCTATGACATTTTTC<br>Reverse: TCGAGAAAAATGTCATAGCTCGGATGTCCAGAATTCTGGACATCCGAGCTATGACAC
