# Supplementary material for: Kidins220 deficiency causes ventriculomegaly via SNX27-retromer-dependent AQP4 degradation
Source: Mol Psychiatry. 2021 May 17;26(11):6411–26. doi: 10.1038/s41380-021-01127-9 (PMC8760065; doi:10.1038/s41380-021-01127-9)
Supplement: Supplementary file 2 — SUPPLEMENTAL MATERIALS AND METHODS [file 41380_2021_1127_MOESM2_ESM.pdf]

Supplemental Materials and Methods

Magnetic resonance imaging.

MRI studies were performed in the High Field MRI and Spectroscopy facility (IIBM, CSIC-UAM) using a Bruker Biospect 7.0-T horizontal-bore system (Bruker Medical Gmbh, Ettlingen, Germany), equipped with a ^1H selective birdcage resonator of 23 mm and a Bruker gradient insert with 90 mm of diameter (maximum intensity 36 G/cm). All data were acquired using a Hewlett-Packard console running Paravision software (Bruker Medical Gmbh) operating on a Linux platform. Anaesthesia was initiated by inhalation of oxygen (1 L/min) containing 3% isoflurane and maintained during the experiment employing a mask and 2% isoflurane in O_2. Animal temperature was maintained at approx. 37°C with a heating blanket. The respiratory rate of the animals was monitored using a Biotrig physiological monitor (SA Instruments, Stony Brook, NY) and the breathing rate was maintained in 60 +/- 40 bpm. T2-weighted (T2-W) spin-echo anatomical images were acquired with a rapid acquisition with relaxation enhancement (RARE) sequence in axial orientations and the following parameters: TR = 2500 ms, TE = 44 ms, RARE factor = 8, Av = 6, FOV = 2.3 cm, acquisition matrix = 256 × 256, slice thickness = 1.00 mm without gap and number of slices = 14. To calculate the ventricular volume of each ventricle in mm^3, the ventricular area was measured in all series of images obtained in MRI-T2W for each animal. Diffusion weighted images (DWI) were acquired with the following parameters: TR = 3500 ms, TE = 27 ms, Av =3, 3 gradient direction, diffusion gradient duration = 3 ms, diffusion gradient separation = 18 ms, b factors = 200, 400 and 1000s/mm^2, FOV: 2.5cm, acquisition matrix = 128 × 128, slice thickness: 1.5mm without gap and number of slices 7. Apparent Diffusion Coefficient (ADC) maps were calculated with a homemade software application written in Matlab (R2007a). The values were extracted

from maps using the region of interest (ROI) with the Image J software (Wayne Rasband, NIH, Bethesda, MD, USA) as described above.

Scanning electron microscopy.

Lateral wall isolated from 2 months-old mice were fixed with 2% glutaraldehyde in PBS pH 7.2–7.4 at 4°C for 4 h and post-fixed with 2% osmium tetroxide in PBS during 2 h in darkness at room temperature. Then, samples were dehydrated through a series of aqueous ethanol solutions (30, 70, 90, 96, and 100% ethanol) and finally dried with liquid CO_2 (critical point at 8°C). Images were obtained as a result of backscattered electrons (BSE), after covering samples with a cycle of evaporation of gold atoms induced by argon in vacuum conditions (0.05–0.07 mbar). SEM images were obtained at the Scan Electron Microscopy facility (Servicio Interdepartamental de Investigación – SIdI – UAM) using a Hitachi S-3000N Scanning Electron Microscope (Chiyoda, Tokyo, Japan).

RNA isolation, reverse transcription and quantitative real-time PCR.

Total RNA from brain periventricular area (PVA) samples or cultured astrocytes of *Kidins220*$^{f/f}$ and WT mice was isolated with RNeasy Mini Kit (Qiagen, The Netherlands) and treated with RQ1 RNase-free DNase (1 U per µg RNA; Promega Corporation, Madison, WI). Total RNA was reverse-transcribed into cDNA, using oligo-dT extension with Super Script III First-Strand Synthesis SuperMix kit from ThermoFisher Scientific (Waltham, Massachusetts, USA) with the amplification protocol 30″ at 95°C + (5″ at 95°C + 5″ at 60°C) × 40 + (5″ at 60°C + 5″ at 95°C). Quantification was performed by real-time PCR using a CFX 384 System (Bio-Rad) in combination with SsoFast Eva Green (Bio-Rad), as per manufacturer's protocol and 1 µl of primer pair was used. Data were analyzed by GenEx 5.3.7 software (Multid

AnaLyses AB). The pairs of primers specific for mouse transcripts of *Aqp4*, *Snx27*, *Vps35, S100β* and *Kidins220* are detailed in Supplementary Table 3. Data were normalized to mice *Gapdh* transcript levels and mRNA abundance was calculated using the $\Delta\Delta C_T$ method and assigned an arbitrarily value of 1 to one control sample.

Plasmids

Lentiviral vectors containing shRNAs to interfere mouse *Kidins220* (shKidins220) and mouse *Snx27* (shSNX27-1, shSNX27-2, shSNX27-3) expression were generated by cloning sh-oligonucleotides into the *Hpa*I and *Xho*I sites of pLentiLox3.7 (pLL3.7; see detailed sequences in Supplementary Table 3). Control shRNA vector (shC) was constructed by introducing oligonucleotides that do not match any known mice transcript. The integrity of these novel constructs was verified by sequencing. SNX27 full length (SNX27-FL), ΔPDZ-SNX27 and mCherry-SNX27 expression vectors were kindly provided by Peter Cullen (University of Bristol, UK). SNX27-FL and ΔPDZ-SNX27 cDNA sequences were subcloned into HindIII and ApaI sites of pEGFP-C3 to generate GFP-SNX27-FL and GFP-ΔPDZ-SNX27 plasmids. GFP-M23-AQP4 expression vector was provided by Mahmood Amiry-Moghaddam (University of Oslo, Norway).

Cell culture, treatment and transfection of primary cortical astrocytes and HEK293T cells.

Mouse cortical astrocytes were prepared from cerebral cortices of 0-2 day-old mice pups according to previously described [1], with slight modifications. After removal of meninges, cerebral cortices were mechanically dissociated in Dulbecco's minimum essential medium and nutrient mixture F-12 (DMEM-F12) completed with 10% foetal bovine serum (FBS) and penicillin (100 U/ml), streptomycin (100 U/ml), all these

reagents from ThermoFisher Scientific. Cellular pellets from each individual pup cortex were obtained by centrifugation at 1000 x g for 5 min, resuspended and plated at a density of 3-5 x 10^5 cells/cm^2 in complete DMEM-F12. Cells were incubated at 37ºC, 5% CO_2 and 95% humidity. After 3 days *in vitro* (DIV), plates were shaken to remove microglia. At DIV12-14 the confluent astrocyte layer was detached with trypsin-EDTA at 37 °C. Resuspended cells were centrifuged at 1000 x g for 5 min and seeded on plates or glass coverslips coated with poly-L-lysine (10 µg/ml) and laminin (4 µg/ml) at a density of 5 x 10^4 cells/cm^2 in complete DMEM-F12. After 2 additional days (DIV14-16) astrocytes were treated with 100 nM bafilomycin A1 or DMSO from Sigma-Aldrich (St Louis, MO, USA) during 16 h. HEK293T cells (purchased from ATCC, tested to be *Mycoplasma*-negative and not further authenticated) were cultured at 37 °C in DMEM supplemented with 10% FBS, 2 mM glutamine and penicillin (100 U/ml), streptomycin (100 U/ml) from ThermoFisher Scientific, in a humidified atmosphere containing 5% CO_2. For transfection, HEK293T cells and DIV12-14 primary astrocytes were seeded at 50-60% confluence. Two days later cells were transfected in Opti-MEM by using 0.7 µl of Lipofectamine 2000® reagent (ThermoFisher Scientific) and 0.7 µg of DNA per 24 multi-well plate, according to the manufacturer's specifications. Cells were fixed and processed for immunofluorescence or washed with PBS to prepare protein extracts for immunoprecipitation experiments.

Lentiviral production and transduction of astrocytic cultures.

Lentiviral suspensions were prepared in HEK293T cells as previously described [2]. Briefly, HEK293T were transfected with lentiviral and packaging vectors using Lipofectamine 2000® reagent and OPTI-MEM media (ThermoFisher Scientific) for 4 h following the manufacturer instructions. Medium was then changed to Iscove´s Modified Dulbecco´s Medium (IMDM) complemented with 5% FBS, 5% horse serum,

22.2 mM glucose, 0.1 mM Glutamax-I, penicillin (100 U/ml), and streptomycin (100 U/ml) (ThermoFisher Scientific). Supernatant was collected after 48 h. For concentration, the viral suspension was first filtered using a Steriflip-HV 0.45-μm filter unit (Millipore, Billerica, MA, USA) and ultracentrifuged at 20,000 rpm for 2 h at 4°C in a SW28 Beckman Coulter rotor. Viral pellets were resuspended overnight at 4°C in PBS. DIV12-14 cultured astrocytes were transduced with concentrated lentiviral suspensions (10^7–10^8 pfu/ml) directly added to the growing media for 7 days.

Water permeability and cell-volume measurements in cultured astrocytes.

Cell-volume changes in WT and *Kidins220*$^{f/f}$ astrocytes were analysed by the calcein-quenching fluorescence method [3]. DIV12-14 cultured astrocytes were grown on glass bottom dishes coated with poly-L-lysine and laminin for 2 days before *in viv*o confocal microscopy analysis. Cells were maintained with the appropriate saline buffer at room temperature and loaded with calcein (ThermoFisher Scientific) for 30 min at 37ºC in the incubator. Calcein dye was excited at 495 nm, and emission was detected at 525 nm. For a steady baseline recording and defining regions of interest (ROIs) around individual cells to collect calcein photometric data, cultures were maintained for 60 s in control saline. The initial average resting fluorescence, expressed in fluorescence units (F), of an individual cell during 60s perfusion in control isotonic medium (300 mOsm) was obtained and normalized to 1.00 (Ft/Ft0) to measure volume-regulatory behavior using ImageJ software (Wayne Rasband, NIH, Bethesda, MD, USA). The hypotonic challenge was rapidly applied by adding water to reach hypotonic solutions (150 mOsm; ΔOsm = 150 mOsm and 75 mOsm; ΔOsm= 225 mOsm), causing cell swelling that was registered as a variation in calcein fluorescence signal at each time point (ΔFt). Means of ΔFt values recorded at 65 s and 170 s after hypotonic challenge were calculated and compared in order to establish water permeability relative to each condition. Maximum change in

fluorescence defined as variation of fluorescence (ΔFtmax) corresponds to the maximum change in the volume after hypotonic challenge exposure. This challenge initiated a regulated volume decrease (RVD) leading to fluorescence recovery toward the control level. The regulatory volume changes were calculated by the mean of Δ(Ft300−Ftmax170s)/Ftmax170s, being Ftmax the maximum level of fluorescence corresponding to maximum change in volume for each condition.

Commercial antibodies. Rabbit polyclonal antibodies were: Kidins220 (Generated at T. Iglesias laboratory [4]), AQP4 (Sigma-Aldrich, St Louis, MO, USA), β-catenin (Cell Signaling Technology, Beverly, MA, USA), GFP (ThermoFisher Scientific) and SNX17 (Proteintech, Manchester, UK). Goat polyclonals were: VPS35 and GFAP (Abcam, Cambridge, UK). Mouse monoclonals were: β-actin, α-tubulin, S100β, γ-tubulin (Sigma-Aldrich), GFAP, FoxJ1 (ThermoFisher Scientific), Kidins220, SNX27 (Abcam) and Rab5 (Synaptic Systems, Goettingen, Germany). Horseradish peroxidase-conjugated anti-rabbit and anti-mouse secondary antibodies were from Santa Cruz Biotechnology and Alexa-Fluor-488, -555, and -647 conjugated anti-rabbit, anti-mouse and anti-goat antibodies were from ThermoFisher Scientific. Detailed information about all the above-mentioned antibodies and dilutions used for the different applications is given in Supplementary Table 2.

Preparation of protein extracts, immunoprecipitation, and immunoblot analysis.

Protein extracts from mice tissue, primary cell cultures or HEK293T cells were prepared in RIPA as previously described [5]. Briefly, cultured cells were lysed in RIPA buffer (25 mM Tris-HCl, pH 7.6, 1% Triton X-100, 0.5% sodium deoxycholate, 0.1% SDS, 150 mM NaCl) with protease and phosphatase inhibitors for 30 min at 4 °C. Lysates were centrifuged for 30 min at 14,000 rpm at 4°C, and resulting supernatant was considered the total lysate soluble fraction. Different mouse tissues were dissected,

frozen in dry ice and homogenized in RIPA buffer as above using potter and Polytron homogenizers. For immunoprecipitation, total lysates were incubated with anti-VPS35 or anti-GFP antibody for 4 h at 4°C, before binding immunocomplexes to protein A sepharose beads for 1 h at 4°C. Beads were washed four times with RIPA buffer before adding Laemli sample buffer. Note that sample boiling was avoided to detect properly AQP4 band by immunoblots. Equal amounts of total lysates or immunoprecipitates were resolved in SDS–PAGE and analysed by immunoblot. Membranes were incubated with different primary and secondary antibodies (see detailed information in Supplementary Table 2) and immunoreactive bands were detected by ECL (PerkinElmer, Waltham, MA USA). Immunoblot images have been cropped for presentation.

Immunofluorescence of cultured astrocytes.

Primary astrocytes grown on coverslips coated with poly-L-lysine and laminin were fixed with 4% PFA with 4% sucrose, permeabilised with 0.1% Triton X-100 in PBS for 5 min and then incubated in blocking solution (5% donkey serum in PBS) for 30 min at room temperature. Coverslips were then incubated for 1 h at room temperature with the appropriate primary antibody in blocking solution followed by the corresponding secondary antibody (see detailed information in Supplementary Table 2). Nuclei were stained with DAPI and coverslips were mounted with Fluoromount-G medium (ThermoFisher Scientific).

Immunohistochemistry of mouse and human brain samples.

Mice were anesthetized by an i.p. injection of pentobarbital and then transcardially perfused with 4% PFA. Brains were removed from mice skull and post-fixed in 4% PFA overnight and cryoprotected in 30% sucrose in PBS for 48 h before freezing.

Complete series of 30 μm-thick coronal sections were obtained by cryostat (Leica Microsystems, Germany). For immunofluorescence floating sections were incubated in blocking solution (5% goat serum, 1% BSA and 0.1% Triton X-100 in 0.1M PBS) for 1 h and incubated in blocking buffer with the appropriate primary antibodies at 4ºC overnight and subsequently incubated 2 h at 4ºC with secondary antibodies. DAPI was used for nuclear staining before mounting slices with Fluoromount-G medium (ThermoFisher Scientific). Fresh tissue was dissected to obtain whole mounts of mouse brain periventricular zone as described [6]. Briefly, the lateral ventricle was dissected from the caudal aspect of the telencephalon, and the hippocampus and septum were removed. The dissected lateral wall was fixed in 4% PFA/0.1% Triton X-100 overnight at 4°C. After staining as described above, the ventricular walls were further dissected from underlying parenchyma as slivers of tissue 200–300 μm in thickness and mounted on a slide with mounting media and a coverslip. For immunohistochemistry floating mouse brain coronal sections were stained free-floating using the biotin–avidin peroxidase method and 3-3′ diaminobenzidine (DAB; Sigma FAST DAB, Sigma-Aldrich) as chromogen. Selected pictures correspond to the rostral region of brain cortex and hippocampus. Endogenous peroxidase was inactivated by incubating sections in 0.3% hydrogen peroxide solution in PBS for 30 min. Brain sections were pretreated for 1 h with 1% BSA, 5% FBS, and 0.2% Triton X-100 in PBS, and subsequently incubated with rabbit polyclonal antibodies against Kidins220. Finally, brain sections were incubated with avidin–biotin complex using Elite Vectastain kit (Vector Laboratories, Burligame, CA, USA). Chromogen reactions were performed with DAB and 0.003% hydrogen peroxide for 10 min before mounting with Prolong medium (ThermoFisher Scientific).

Paraffin embedded human post-mortem brain 15-µm sections were processed, treated with EnVision FLEX (Dako, K8004, Denmark) for antigen retrieval during 20 min at 90ºC and incubated in blocking solution (10% donkey serum and 0.2% Triton X-100 in PBS) for 1 h. For double immunofluorescence analysis, sections were incubated with primary antibodies for 2 days in blocking solution followed by washes in PBS and incubation with secondary antibodies in the same solution. Then, slices were rinsed in PBS and nuclei were stained with DAPI. Sections were treated with a saturated solution of Sudan Black B (Sigma-Aldrich) for 15 min to block autofluorescence of lipofuscin granules. Sections were later washed and mounted with Prolong medium (ThermoFisher Scientific). Specific regions of interest (ROI) boundaries were defined at the ependymal cells lining lateral ventricles, whose nuclei were stained with DAPI, and Kidins220 or AQP4 immunofluorescence intensity signal was quantified. Quantification studies were performed at 9 different ROIs defined at the ependymal barrier area of each individual.

Image acquisition.

Confocal microscopy images were acquired using plan-apochromatic objectives in an inverted Zeiss LSM 710 laser-scanning microscope (Zeiss, Germany). To avoid crosstalk between channels, sequential scanning mode was used. All images shown correspond to maximum projection of serial sections, except those used for colocalization analysis. Pictures were processed with Zen 2009 (Carl Zeiss MicroImaging), Adobe Photoshop CS (Adobe Systems Inc.), and ImageJ 1.47d (NIH) software. DAB staining bright field images were captured in a Nikon Eclipse 90i microscope, using a Digital Sight DS-QiMc camera and NIS-Elements BR 3.0 software (Nikon, Japan).

Quantitative and statistical analysis.

Immunoblot signals were quantified by densitometric analysis with ImageJ 1.47d software (NIH) and normalized using β-actin, α-tubulin or GFAP band signal intensities. Data were expressed relative to values obtained in their respective untreated controls or WT mice or astrocytes. For immunofluorescence quantitative analysis of mice and human brain samples, fluorescence intensity of specific antibodies was measured at the ependymal barrier of lateral ventricles using ImageJ 1.47d software (NIH). Specific ROIs were defined in each brain section at the ependymal barrier boundaries, identified by DAPI staining of ependymal cell nuclei. In those regions, fluorescence signal above a certain threshold, stablished as background, was quantified. In the case of human brain necropsies, nine randomly selected ROIs were used for the analysis. For murine samples, three independent sections per animal were used, each containing 3 ROIs. In both cases, results were expressed as the average fluorescence intensity, and relative to values detected in control samples, arbitrarily assigned a value of 100. In cultured astrocytes fluorescence intensity of specific antibodies was measured in 60-80 GFAP+ cells for each condition or 40-80 GFP+ cells in those astrocytic cultures transfected or transduced with GFP-expressing plasmids. Analyses of significant differences between means were carried out using unpaired Student's *t* test, one-way or two-way ANOVA tests with Tukey multiple comparison test, when appropriated according to the data distribution, as specified in each figure legend. Variance was comparable between groups in experiments described throughout the manuscript. A Grubbs' test, also called the ESD method (extreme studentized deviate) was performed to determine whether one of the samples or animals in each group was a significant outlier from the rest, with a significant level of $\alpha=0.05$. No statistical method was used to predetermine sample size. Sample sizes were based on

previously published experiments by others and us, and on our own unpublished data. The experiments were not randomized. The investigators were not blinded to allocation during experiments or outcome assessment. Results are shown as mean ± s.e.m. and the number of experiments carried out with independent primary astrocytic cultures and animals (*n*) is shown in each figure legend. Statistical significance was assigned at *0.01<P<0.05, **0.001<P<0.01 and ***P<0.001; n.s, not significant.

References for Supplemental Materials and Methods section:

1. Schildge S, Bohrer C, Beck K, Schachtrup C. Isolation and culture of mouse cortical astrocytes. *J Vis Exp* 2013; (71).

2. Pose-Utrilla J, Garcia-Guerra L, Del Puerto A, Martin A, Jurado-Arjona J, De Leon-Reyes NS *et al.* Excitotoxic inactivation of constitutive oxidative stress detoxification pathway in neurons can be rescued by PKD1. *Nat Commun* 2017; 8(1): 2275.

3. Benfenati V, Caprini M, Dovizio M, Mylonakou MN, Ferroni S, Ottersen OP *et al.* An aquaporin-4/transient receptor potential vanilloid 4 (AQP4/TRPV4) complex is essential for cell-volume control in astrocytes. *Proc Natl Acad Sci U S A* 2011; 108(6): 2563-2568.

4. Lopez-Menendez C, Simon-Garcia A, Gamir-Morralla A, Pose-Utrilla J, Lujan R, Mochizuki N *et al.* Excitotoxic targeting of Kidins220 to the Golgi apparatus precedes calpain cleavage of Rap1-activation complexes. *Cell Death Dis* 2019; 10(7): 535.

5. Lopez-Menendez C, Gamir-Morralla A, Jurado-Arjona J, Higuero AM, Campanero MR, Ferrer I *et al.* Kidins220 accumulates with tau in human Alzheimer's disease and related models: modulation of its calpain-processing by GSK3beta/PP1 imbalance. *Hum Mol Genet* 2013; 22(3): 466-482.

6. Porlan E, Marti-Prado B, Morante-Redolat JM, Consiglio A, Delgado AC, Kypta R *et al.* MT5-MMP regulates adult neural stem cell functional quiescence through the cleavage of N-cadherin. *Nat Cell Biol* 2014; 16(7): 629-638.
